# Use of Whole Genome Sequencing for Diagnosis and Discovery in the Cancer Genetics Clinic

**DOI:** 10.1016/j.ebiom.2014.12.003

**Published:** 2014-12-03

**Authors:** Samantha B. Foley, Jonathan J. Rios, Victoria E. Mgbemena, Linda S. Robinson, Heather L. Hampel, Amanda E. Toland, Leslie Durham, Theodora S. Ross

**Affiliations:** a Department of Internal Medicine, UT Southwestern Medical Center, Dallas TX, 75390, USA; b Sarah M. and Charles E. Seay Center for Musculoskeletal Research, Texas Scottish Rite Hospital for Children, Dallas, Texas 75219, USA; c Department of Pediatrics, McDermott Center for Human Growth and Development, and Department of Orthopaedic Surgery, UT Southwestern Medical Center, Dallas, Texas 75390, USA; d Department of Cancer Genetics, UT Southwestern Medical Center, Dallas, TX, 75390, USA; e Department of Human Genetics, Ohio State University, Columbus, OH, 43210, USA

**Keywords:** Cancer genetics, BRCA1/2, Whole-genome sequence, ClinVar, Pathogenic variants, Single nucleotide variant

## Abstract

Despite the potential of whole-genome sequencing (WGS) to improve patient diagnosis and care, the empirical value of WGS in the cancer genetics clinic is unknown. We performed WGS on members of two cohorts of cancer genetics patients: those with *BRCA1/2* mutations (n = 176) and those without (n = 82). Initial analysis of potentially pathogenic variants (PPVs, defined as nonsynonymous variants with allele frequency < 1% in ESP6500) in 163 clinically-relevant genes suggested that WGS will provide useful clinical results. This is despite the fact that a majority of PPVs were novel missense variants likely to be classified as variants of unknown significance (VUS). Furthermore, previously reported pathogenic missense variants did not always associate with their predicted diseases in our patients. This suggests that the clinical use of WGS will require large-scale efforts to consolidate WGS and patient data to improve accuracy of interpretation of rare variants. While loss-of-function (LoF) variants represented only a small fraction of PPVs, WGS identified additional cancer risk LoF PPVs in patients with known *BRCA1/2* mutations and led to cancer risk diagnoses in 21% of non-BRCA cancer genetics patients after expanding our analysis to 3209 ClinVar genes. These data illustrate how WGS can be used to improve our ability to discover patients' cancer genetic risks.

## Highlights

• We used WGS to analyze the germline variations of 258 cancer genetics patients.
• To interpret variants, *BRCA* mutation carrier genomes were used as controls for patients that did not have *BRCA* mutations.
• The numbers of genetic diagnoses were increased when we expanded our focus to all genes annotated in the *ClinVar* database.
• This study investigates the pitfalls and the potential for diagnosis and discovery using whole-genome germline sequencing.

## 1 Introduction

With the rapid development and decreasing cost of whole-genome sequencing (WGS) technologies, this genetic testing tool will soon be readily available for common use in the laboratory and clinic (Collins and Hamburg, 2013). Though the research community is rejoicing in the newfound ability to use WGS to investigate patients' genomes in better detail, clinicians are more cautious. The unknown, but potentially significant, burden of delivering genetic results of uncertain value to patients weighs heavily on practitioners, such as oncologists, cardiologists and neurologists.

WGS, and whole-exome sequencing, have already been used to provide genetic diagnoses that inform clinical care (Worthey et al., 2011, Bainbridge et al., 2011, Rios et al., 2010). These early successes in individual patients prompted expanded studies to investigate a more general use of WGS in clinical settings (Saunders et al., 2012). Indeed the growing adoption of WGS in the clinic and the potential to positively impact patient care contributed, at least in part, to the UK100K Project, an effort by the Department of Health (United Kingdom) to provide high-coverage WGS for clinical interpretation in 100,000 participants focusing initially on rare diseases, cancer and infectious disease (www.genomicsengland.co.uk). Therefore, we sought to investigate the value of WGS in two cohorts of cancer genetics patients to begin to address the challenges associated with the identification and clinical interpretation of WGS and potentially pathogenic variants (PPVs) in the genetics clinic.

Although patients with a family history of cancer are currently evaluated with single-gene or gene panel tests, it is not clear whether WGS will replicate these findings or potentially increase the rate of identification of genetic risk factors. In this study, we modeled a scenario where WGS replaced gene-specific testing in 176 *BRCA1/2*-carriers and 82 non-*BRCA* patients from our cancer genetics clinics. We sought to examine whether WGS could be easily and quickly mined to identify PPVs highly likely to increase cancer risk as well as potentially expand WGS to assess genetic risk for non-cancer conditions.

## 2 Methods

### 2.1 Participants

Individuals from the cancer genetics clinics of the University of Texas Southwestern Medical Center (UTSW) and the Ohio State University (OSU) cancer genetics programs were recruited to the study following informed consent approved by the Institutional Review Boards of both institutions. Only unrelated individuals were included in this study. Blood samples were obtained and de-identified. Subsequent genetic results were not returned to participants.

### 2.2 *Whole-Genome* Sequencing and Variant Analysis

WGS of DNA was performed by Complete Genomics Inc. (Mountain View, CA, USA) as previously described (Drmanac et al., 2010). Sequence analysis and variant detection were performed by Complete Genomics Inc., as well. Variant analysis was performed as previously described (Soyombo et al., 2013); however, variant quality measures were investigated to determine appropriate quality control parameters for identifying high-quality PPVs from WGS. To determine the variant quality score threshold, we measured genotype concordance between individual-matched WGS from lymphoblast and fibroblast samples from seven individuals, which are expected to have minimal discrepancies. Genotype concordance was measured using quality score thresholds ranging from 50 to 100 (Supplementary Fig. 1). Single nucleotide variants (SNVs) with quality scores less than 100 for both alleles were excluded, resulting in an average SNV genotype concordance rate of 98.88%. Because of the systematically lower concordance and higher error rate for detecting insertions and deletion (indels) compared to SNVs, indel concordance was measured using quality scores ranging from 0 to 300 (Supplementary Fig. 2). Indels with quality scores greater than 150 were included in the study, resulting in an average genotype concordance rate of 97.14%, although an average 63.07% of indels originally identified by Complete Genomics were excluded.

While numerous aspects of WGS performance are considered and represented in the variant quality score provided by Complete Genomics, individual quality parameters may improve WGS specificity and sensitivity, as are commonly used by other sequencing technologies. These include, but are not limited to, overall sequence depth, sequence coverage at variant positions, variant allele fraction, individual read quality and mapping quality, and read directionality for paired-end reads. Though these measures were not individually included in the analysis of WGS from Complete Genomics, the quality score procedure used here was previously shown to improve concordance to orthogonal validation by Sanger sequencing (Soyombo et al., 2013). All sequenced genomes were mapped to the human reference sequence (b37) and analyzed using Complete Genomic's software (version 2.4). A summary of sequencing statistics (coverage, amount of sequence, total variants and genome-wide QC measures) is reported per sample in Supplementary Table 1.

Potentially pathogenic variants (PPVs) were determined primarily by population frequency using the Exome Variant Server (ESP6500) and a population of HapMap individuals sequenced by Complete Genomics. To simplify detection of PPVs, nonsynonymous variants with frequency less than 1% in both the ESP6500 and HapMap datasets were considered potentially pathogenic. Loss-of-function (LoF) nonsynonymous variants were defined as SNVs predicted to create a premature truncation (nonsense), alter canonical splicing (disrupt), alter the initiating methionine of the protein (misstart), or alter the final stop codon of the transcript (nonstop). LoF indels included only those resulting in a frameshift and did not include in-frame deletions and insertions.

### 2.3 Statistical Analyses

All statistical analyses were performed using the R statistical framework. 95% confidence intervals were determined from the binomial probability. Fisher's exact test was used to test for significant differences between WGS and published gene-panel sequencing methods (Kurian et al., 2014).

## 3 Results

### 3.1 WGS Confirms Clinically Diagnosed BRCA1/2 Mutations

WGS was performed on a series of patients from the cancer genetics clinic that included those found to have *BRCA1* (n = 88; Supplementary Table 2) or *BRCA2* (n = 88; Supplementary Table 3) mutations, as well as those that were not carriers of a *BRCA1/2* mutation (n = 82; Supplementary Table 4). The genomes of the 176 unrelated *BRCA*-carriers at high risk for breast and ovarian cancer were first investigated to determine if WGS confirmed the clinically-diagnosed mutations (Myriad Genetics).

Similar to previous reports (Worthey, 2013), PPVs were defined as rare (less than 1% frequency in the ESP6500 and HapMap samples sequenced using the same technology) nonsynonymous variants. Because of the variability provided by different computational methods, *in silico* predictions of variant pathogenicity were not used to evaluate missense PPVs. Finally, due to technical limitations in detection of copy number variants (CNVs) and functional annotation of intronic intervening sequence (IVS) variants, these variants were not considered in our WGS analysis of PPVs. After applying quality control measures (see Methods), WGS identified the majority of clinically-diagnosed *BRCA1* and *BRCA2* mutations. Of the 75 patients with *BRCA1* mutations for which our method was expected to detect the clinically-diagnosed PPV, WGS detected 89.3% of the *BRCA1* mutations; the remaining eight mutations were identified in the WGS data but at lower quality (Supplementary Table 5). Among 88 patients with *BRCA2* mutations, WGS confirmed 88.6% of the *BRCA2* mutations; the remaining ten mutations were identified by WGS but again at lower quality (Supplementary Table 5). In sum, WGS in this cohort detected all *BRCA1/2* mutations expected to be identified by our WGS approach, although limitations in sequence quality prevented confident reporting of ~ 12% of *BRCA1/2* PPVs. We expect this result to be a lower-bound, as sequencing technologies and computational methods continue to improve. Indeed, 16 of 18 (89%) low quality *BRCA1/2* variants were indels, and indels were previously reported to be poorly sequenced using this WGS method (Drmanac et al., 2010). WGS results are summarized for all *BRCA1/2* mutations in Supplementary Tables 6 and 7.

### 3.2 WGS Detects Cancer-risk PPVs in *BRCA1/2* Patients

To model clinical WGS in our patients, we evaluated 163 clinically-relevant genes (Table 1) to identify PPVs in the BRCA1/2 cohort. These genes included cancer genes evaluated on commercial cancer-susceptibility gene panels, genes selected by Dorschner et al. (2013) as genes that might impact reproductive decision making (e.g. carrier-status reporting) as well as genes initially recommended for reporting by ACMG (Green et al., 2013). In total, the initial gene panel represented 135 dominant, 24 recessive and 4 X-linked disease-associated genes.

**Table 1:** 163 disease-gene panel.

Dominant	Dominant (cont.)	Dominant (cont.)	Recessive	X-linked
*ACTA2*^a^	*HIP1*	*PROC*	*ATP7B*	*DMD*
*ACTC1*^a^	*HIP1R*	*PROS1*	*BCHE*	*EMD*
*ACVRL1*	*HMBS*	*PRSS1*	***BLM***	*GLA*^a^
***APC***^a^	*HOXB1*	***PTCH1***	*CASQ2*	*OTC*
*APOB*^a^	***HOXB13***	***PTEN***^a^	*CFTR*	–
***ATM***	*HTT*	***RAD50***	*COQ2*	–
***ATR***	***JAK2***	***RAD51***	*COQ9*	–
***BAP1***	*KCNE1*	***RAD51C***	*CPT2*	–
***BARD1***	*KCNE2*	***RAD51D***	*F5*	–
***BMPR1A***	*KCNE3*	***RAS***	*GAA*	–
***BRCA1***^a^	*KCNH1*	***RB1***^a^	*HAMP*	–
***BRCA2***^a^	*KCNH2*^a^	*RBBP8*	*HFE*	–
***BRIP1***	*KCNJ2*	*RBM20*	*HFE2*	–
*CACNA1C*	*KCNQ1*^a^	***RET***^a^	*IDUA*	–
*CACNA1S*^a^	***KDR***	*RYR1*^a^	*INPP5B*	–
*CACNB2*	***KIT***	*RYR2*^a^	*LDLRAP1*	–
***CDC73***	*LDLR*^a^	*SCN1B*	*PAH*	–
***CDH1***	*LMNA*^a^	*SCN3B*	*PCBD1*	–
***CDK4***	***MEN1***^a^	*SCN5A*^a^	*PTS*	–
***CDKN1A***	***MET***	***SDHAF2***^a^	*QDPR*	–
***CDKN2A***	***MITF***	***SDHB***^a^	*SERPINA1*	–
***CDKN2B***	***MLH1***^a^	***SDHC***^a^	*SLC25A13*	–
***CHEK1***	***MLH3***	***SDHD***^a^	*SLC37A4*	–
***CHEK2***	***MRE11A***	*SERPINC1*	*SLC7A9*	–
*CNBP*	***MSH2***^a^	*SGCD*	–	–
*COL3A1*^a^	***MSH6***^a^	*SMAD3*^a^	–	–
***CREBBP***	***MUTYH***^a^	***SMAD4***	–	–
*DMPK*	*MYBPC3*^a^	***SMARCB1***	–	–
*DSC2*^a^	*MYH11*^a^	***SMO***	–	–
*DSG2*^a^	*MYH7*^a^	***STK11***^a^	–	–
*DSP*^a^	*MYL2*^a^	*TGFB3*	–	–
***EGFR***	*MYL3*^a^	*TGFBR1*^a^	–	–
***ELAC2***	*MYLK*^a^	*TGFBR2*^a^	–	–
*ENG*	***NBN***	*TMEM43*^a^	–	–
***EPCAM***	***NF1***	*TNNI3*^a^	–	–
***FAM175A/Abraxas***	***NF2***^a^	*TNNT2*^a^	–	–
*FBN1*^a^	***NTRK1***^a^	***TP53***^a^	–	–
***FH***	***PALB2***	***TP53BP1***	–	–
***FLCN***	*PCSK9*^a^	*TPM1*^a^	–	–
***GALNT12***	***PDGFRA***	***TSC1***^a^	–	–
*GCH1*	*PKP2*^a^	***TSC2***^a^	–	–
***GEN1***	*PLN*	***VHL***^a^	–	–
*GPD1L*	***PMS2***^a^	***WT1***^a^	–	–
***GREM1***	*PRKAG2*^a^	***XRCC2***	–	–
*HCN4*	*PRKAR1A*	***XRCC3***	–	–

Bold = cancer-associated genes.

a ACMG genes.

**Fig. 1:**
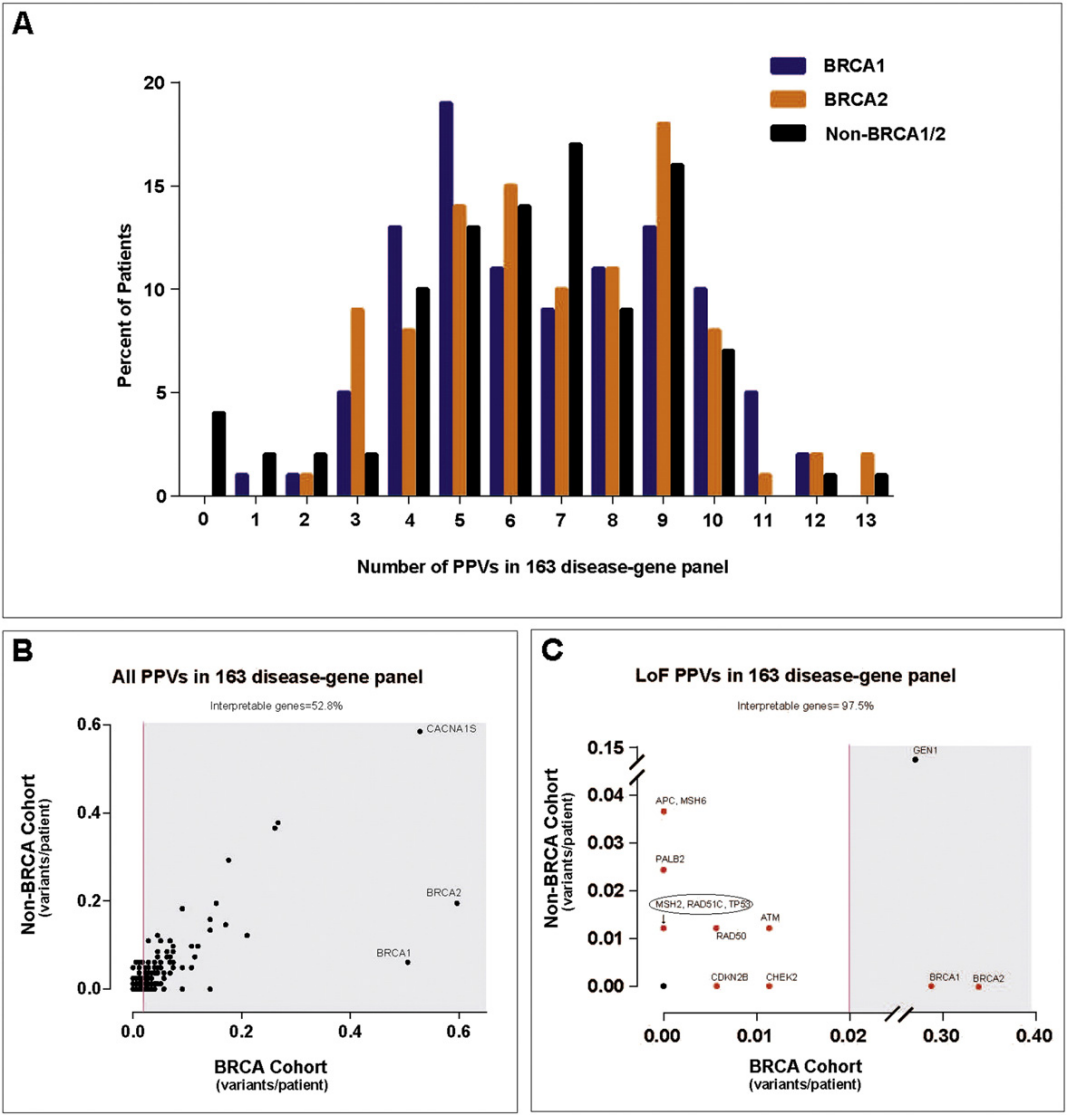
Analysis of potentially pathogenic variants (PPVs) in 163 disease genes. (A) Histogram distribution of the number of PPVs from WGS analysis of 163 genes is shown. Each clinic cohort is shown, including patients with either clinically-diagnosed *BRCA1* mutations (blue), *BRCA2* mutations (orange) or without *BRCA1/2* mutations (black). (B, C) “Gene variance plot” of carrier burden (average number of PPVs per individual) for (B) all PPVs in the 163 disease-gene panel or (C) loss-of-function (LoF) PPVs in the 163 disease-gene panel. Genes harboring PPVs in at least 2% of the BRCA1/2 cohort are shown in the shaded region. Those genes not in the shaded region were considered “clinically interpretable” by WGS. One dot can represent several genes. For instance the black dot at zero in both populations represents many genes lacking PPVs in both cohorts. Genes shown in red are those that harbored cancer-risk PPVs in patients, also listed in Table 2, Table 3.

WGS identified 1207 PPVs in the 176 patient genomes with *BRCA* mutations, representing 695 unique variants. Of these, most were missense variants and 46.33% of all PPVs were novel and not present in the ESP6500 database (Supplementary Table 8). On average, WGS identified 6.8 and 6.9 PPVs per patient in the *BRCA1*- and *BRCA2*-carrier cohorts, respectively (Fig. 1A). Among the 163 genes, the number of PPVs per gene varied greatly (Supplementary Tables 9 and 10). Similar gene variance results were obtained for *BRCA1*- or *BRCA2*-carrier cohorts evaluated separately (Supplementary Tables 11 and 12). As expected, when restricting our analysis to only loss-of-function (LoF) PPVs (see Methods), the number of variants decreased dramatically. This indicates that a majority of nonsynonymous PPVs in the cancer genetics clinic will be classified as variants of unknown significance (VUS), reinforcing the challenge of expanding the scope of clinical WGS (Biesecker, 2012); namely, the interpretation of novel nonsynonymous (missense) PPVs.

To begin to address this, we reviewed each of the 695 PPVs for published evidence of pathogenicity or association with disease. In many individuals for whom family members were recruited, as has been found by Johnston et al. and others in their evaluation of next generation sequencing (Johnston et al., 2012), we found no evidence for co-segregation of the variant with disease or found inconsistencies in their association with disease in the literature. For example, WGS identified a patient with a missense variant (p.Asn1978Ser) in *CREBBP* previously reported as pathogenic and diagnostic for autosomal dominant Rubenstein Taybi Syndrome (RTS) (Bartsch et al., 2005, Roelfsema and Peters, 2007). Subsequently, four additional individuals in our *BRCA1/2* cohort with the same variant were identified and confirmed by Sanger sequencing. Detailed family history as well as DNA samples from several family members of one index patient with this *CREBBP* variant were available, and the patient and her family members that carried the variant had no symptoms consistent with a RTS diagnosis (Supplementary Fig. 3). In a second example, WGS identified a missense variant (p.Asn29Ile) in *PRSS1* considered pathogenic by clinical laboratories (e.g. Ambry Genetics) as it has previously been associated with hereditary pancreatitis and subsequent pancreatic cancer (Whitcomb, 2013, Sanchez-Ramirez et al., 2012, Gorry et al., 1997). A total of nine individuals were identified with this same variant and confirmed by Sanger sequencing, and no *PRSS1*-variant carrying individual reported pancreatic problems. Further investigation of these patients' pedigrees showed that one individual's family history supported the WGS result: the patient's father, who was unavailable for testing but presumed to transmit the variant (her mother lacked the *PRSS1* variant) died of pancreatic cancer at age 53 (Supplementary Fig. 4). While WGS has potential to expand the breadth of disease risk reported to individual patients, it is evident that clinical interpretation of WGS results, particularly for missense variants, requires detailed medical histories and results from extended family members to have confident diagnoses for appropriate genetic counseling. Importantly, previously reported disease associations should be met with caution, not considered to be equivalent to *causation*, and be interpreted in light of available family medical history.

### 3.3 Comparison of WGS Results in Two Cancer Genetics Clinic Cohorts

Because clinical WGS is likely to report an overwhelming number of VUSs (Domchek et al., 2013, Yorczyk et al., 2014), we sought to evaluate the frequency of PPVs in individual genes in different clinic populations. We compared the WGS results from the *BRCA1/2*-carrier cohort (n = 176) to the non-*BRCA* cancer genetics clinic cohort (n = 82) using a “gene variance” analysis. The two cohorts served as reciprocal controls since the majority of the cancers present in the BRCA1/2 cohort presumably resulted from the patients' pathogenic *BRCA1/2* mutations, while the non-BRCA cohort had either negative tests for *BRCA1/2* mutations or cancers not associated with *BRCA1/2*. Because PPVs were selected with less than 1% allele frequency, heterozygous individuals are expected to occur in less than 2% of patients in the BRCA1/2 “control” population. Using this method, we concluded that genes reported as harboring PPVs in less than 2% of this cohort are “clinically interpretable” using WGS and not likely to result in numerous false-positive PPVs reported to patients. As such, many “clinically interpretable” genes had no PPVs in either cohort.

This “gene variance” analysis allowed us to calculate and visualize the percentage of clinically interpretable genes (excluding *BRCA1* and *BRCA2*), defined as genes with less than 2% of individuals carrying PPVs in the “control” BRCA1/2 cohort, using different approaches to define PPVs. Including all nonsynonymous PPVs, only 52.8% of genes were considered clinically interpretable (Fig. 1B). Interestingly, 6% and 20% of the non-BRCA cohort had PPVs in *BRCA1* and *BRCA2*, respectively, and both genes were among the most variable genes we investigated. We note that these PPVs were previously deemed benign with clinical testing (Myriad Genetics) in patients for whom their cancer could have been associated with *BRCA1/2* mutations. Additionally, while 67.3% and 66.2% of PPVs in the *BRCA1* and *BRCA2* genes in the BRCA1/2 cohort, respectively, were predicted LoF variants, none of the four *BRCA1* or 16 *BRCA2* variants in the non-BRCA cohort were predicted LoF variants. These results highlight the challenge, even for commonly-tested genes, of clinical interpretation for rare missense variants detected by WGS. It also underscores the increased specificity of clinical WGS by investigating LoF variants, the majority of which are likely to have similar functional consequences regardless of the genetic heterogeneity of individual genes. The lack of clinical specificity among missense PPVs was evident, as there was a correlation of PPV frequency with coding region size in the *BRCA1/2* (r^2^ = 0.61) and non-BRCA (r^2^ = 0.5217) cohorts (Supplemental Fig. 5A, C). When restricting the analysis to LoF PPVs the correlation with size was lost, suggesting LoF PPVs may be more associated with the patient's clinical presentation than the gene's background mutation rate (Supplemental Fig. 5B, D).

When restricted to LoF PPVs, the percent of clinically interpretable genes increased to 97.5%, and PPVs clinically diagnosed as pathogenic mutations by Myriad Genetics in the *BRCA1* and *BRCA2* genes were now specific to the BRCA1/2 cohort (Fig. 1C). Restricting clinical interpretation to LoF variants has three advantages. First, LoF variants are more likely to be associated with human disease and may be enriched for diagnostic variants compared to other nonsynonymous variants. Second, restricting results to LoF variants greatly reduces the number of PPVs returned to patients and may simplify interpretation of reported variants, for both the clinician and patient. However, we recognize that the increase in the percent interpretable genes is due mostly to the increase in the number of genes without PPVs in both cohorts and that this may also increase the false-negative rate of our simplified approach. Although this is reasonable in the research setting, false-negative test results in the clinical setting are of little benefit and methods are needed to improve the sensitivity and specificity of WGS for clinical diagnosis. And last, although a frequency less than 1% is a commonly used threshold for defining PPVs, rare variants are more likely to be population specific and a universal 1% threshold may not be appropriate for all ethnicities. However, we did investigate alternative frequency thresholds and found that this approach performed best in our population (Supplementary Table 13). Even still, some genes reported a high frequency of LoF PPVs in both populations. *GEN1* harbored a single novel deletion resulting in a predicted frame-shift variant that was frequently present in both cohorts. This variant is likely a sequencing artifact because of its frequency in both populations and our inability to confirm it by Sanger sequencing (data not shown). This may foreshadow future difficulties for clinical interpretation of *GEN1* LoF variants (indels) by next-generation sequencing methods.

After excluding *GEN1* from the analysis, the WGS data identified six individuals in the BRCA1/2 cohort with LoF PPVs in four dominant cancer-associated genes, in addition to their clinically diagnosed *BRCA1/2* mutation (Table 2 and Fig. 1C). Therefore, WGS identified additional predicted cancer risk mutations in *BRCA1/2*-carrier patients that were missed using standard clinical methods. Interestingly, one individual (BRCA2.65) with a history of melanoma had a nonsense variant in *CDKN2B* (p.Glu35*) that, to our knowledge, is the first reported individual with a germline LoF variant in *CDKN2B* (also known as *p15^Ink4b^*); no germline *CDKN2B* LoF variants are reported from exome sequencing of 6503 individuals (ESP6500). The knowledge that patients carry additional deleterious cancer-predisposing mutations would be of definite use for counseling and then screening of the patient's family members.

**Table 2:** Potentially pathogenic LoF variants in cancer-associated genes in *BRCA1/2*-carriers.

Patient	Gene	Variant^a^	Patient cancer history^b^
*Discovered cancer gene mutations (163 disease-gene panel)*^c^			
BRCA1.60	CHEK2	c.573+1G>A	Breast-47, 49
BRCA1.61	ATM	**p.Glu2290**⁎	Unaffected-24
BRCA2.7	RAD50	c.3G>A	Breast-49
BRCA2.13	ATM	p.Glu1978⁎	Breast-28, 39, 55; Skin-49
BRCA2.65	CDKN2B	p.Glu35⁎	Melanoma-50
BRCA2.93	CHEK2	p.Gln20⁎	Breast-37, 61; Ovarian-56

*Discovered cancer gene mutations (ClinVar)*^c^			
BRCA1.48	ERCC3	p.Arg109⁎	Breast-39; Skin-61, 66
BRCA1.73	DLEC1	c.2436-2A>G	Breast-32
BRCA1.74	FANCC	p.Arg548⁎	Breast-42, 53, 55

a The one indel variant is shown in bold.

b Age at diagnosis or current age of unaffected.

c Discovered variants were identified by WGS and not tested by clinical genetic testing performed as standard of care.

⁎ Stop gain mutation.

Analysis of LoF PPVs in genes that cause diseases other than cancer identified genetic risks in 19 of the 176 (10.8%) *BRCA1/2*-carriers (Supplementary Table 14). For example, WGS identified a heterozygous nonsense variant (p.Gly542*) in the *CFTR* gene. Although homozygous or compound heterozygous mutations in the *CFTR* gene cause cystic fibrosis, there is little evidence to date that the knowledge would be used for anything other than potentially prenatal counseling. As expected, WGS has the potential to provide additional genetic risk assessments for conditions other than the primary diagnosis (cancer), and these results will require genetic counseling.

### 3.4 WGS Detects Cancer-risk PPVs in Non-*BRCA* Patients

We next asked whether WGS provided added value in the cohort of 82 unrelated non-BRCA patients (Supplementary Table 4). This cohort is representative of our clinic census with the exception of removing *BRCA1/2*-carriers individuals for the first cohort. Using the same model as described above, the cohort averaged 6.4 PPVs in the selected 163 genes (Fig. 1A; Supplementary Tables 15 and 16), and we subsequently focused only on LoF PPVs, which averaged 0.4 LoF PPVs per individual (Fig. 1C). Excluding *GEN1*, WGS identified LoF PPVs in 14 genes in 18 of 82 (22%) non-*BRCA* individuals. The LoF variants represented only 6% of all PPVs identified by WGS in the 163 genes. Importantly, of the 13 individuals with LoF PPVs in cancer-associated genes, 11 were in genes implicated in the individual's primary cancer diagnosis and two provided a likely genetic diagnosis based on family history (Table 3). WGS also detected LoF PPVs in cancer genes beyond tests performed as standard of care for this population. Two individuals (UTSW9 and UTSW36) harbored nonsense mutations in *PALB2*, which has recently been reported to result in an elevated risk for breast cancer similar to *BRCA2* mutations (Antoniou et al., 2014). A nonsense mutation in *RAD51C* was identified in another individual (UTSW13) who was originally tested in the clinic for an *FH* mutation (WGS identified this mutation as well). Again, this illustrates the potential of WGS and multi-gene panels to identify co-occurring cancer gene mutations (e.g. *RAD51C* and *FH*) that pose genetic risks (breast/ovarian cancer) beyond the patient's primary diagnosis (leiomyoma). WGS is an unbiased approach to identify unanticipated risk factors, and the entirety of genetic information may prove useful as additional disease-causing genes are discovered. WGS in the clinical setting will require extensive pre-test genetic counseling.

**Table 3:** WGS identifies potentially pathogenic LoF variants in cancer-associated genes in the non-*BRCA* cohort.

Patient	Gene	Variant^d^	Cancer history^a^	Family cancer history
*Confirmed cancer gene mutations*^b^				
UTSW3	TP53	p.Arg196⁎	Adrenocortical-1	Breast, lung, prostate
UTSW12	APC	**p.Asp170Valfs**⁎**4**	Colon polyposis-51	None
UTSW13	FH	**p.Gln185Leufs**⁎**18**	Leiomyoma-33	Breast, colon, leiomyoma, RCC
UTSW16	APC	**p.Arg1920Glufs**⁎**50**	Colon polyposis-53	None
UTSW31	MSH6	**p.Phe569Hisfs**⁎**7**	Colon-52	Endometrial
UTSW32	MSH6	p.Arg911⁎	Colon-40	None
UTSW38	FH	**p.Gln185Leufs**⁎**18**	Papillary RCC-48	Breast, leiomyoma
UTSW44	MSH2	p.Arg389⁎	Unaffected-27	Colon
UTSW53	MSH6	**p.Lys1013Ilefs**⁎**3**	Unaffected-30	Colon
UTSW55	APC	**p.Glu1309Aspfs**⁎**4**	Colon polyposis-26	Colon
UTSW78	ATM	**p.Val835Serfs**⁎**7**	Breast-54, 56	Brain, breast, colon, lung, prostate
UTSW78	RAD50	**p.Thr109Asnfs**⁎**20**	Breast-54, 56	Brain, breast, colon, lung, prostate

*Discovered cancer gene mutations (163 disease-gene panel)*^c^				
UTSW9	PALB2	p.Tyr1183⁎	DLCBL-41	Breast, lung, multiple myeloma, RCC
UTSW13	RAD51C	p.ArgR193⁎	Leiomyoma-33	Breast, colon, leiomyoma, RCC
UTSW36	PALB2	p.Glu27⁎	Vulvar-64; Breast-68	Breast, pancreatic, prostate, stomach

*Discovered cancer gene mutations (ClinVar)*^c^				
UTSW22	FANCM	p.Gln1701⁎	Breast-48, 56; Pancreatic-58	Breast, colon, ovarian, prostate, stomach
UTSW51	FANCM	p.Gln1701⁎	Unaffected-40	Brain, lung
UTSW76	ERCC3	p.Arg109⁎	Breast-47	Breast, colon, endometrial, prostate, thyroid
UTSW82	FANCA	p.Glu288⁎	Breast-61; Renal oncocytoma-66	Breast, colon, prostate

Abbreviations: DLCBL, diffuse large B-cell lymphoma; RCC, renal cell carcinoma.

a Age at diagnosis or current age of unaffected.

b Confirmed variants were previously identified by clinical genetic testing performed as standard of care.

c Discovered variants were identified by WGS and not tested by clinical genetic testing performed as standard of care.

d Indel variants are shown in bold.

⁎ Premature stop gain.

**Fig. 2:**
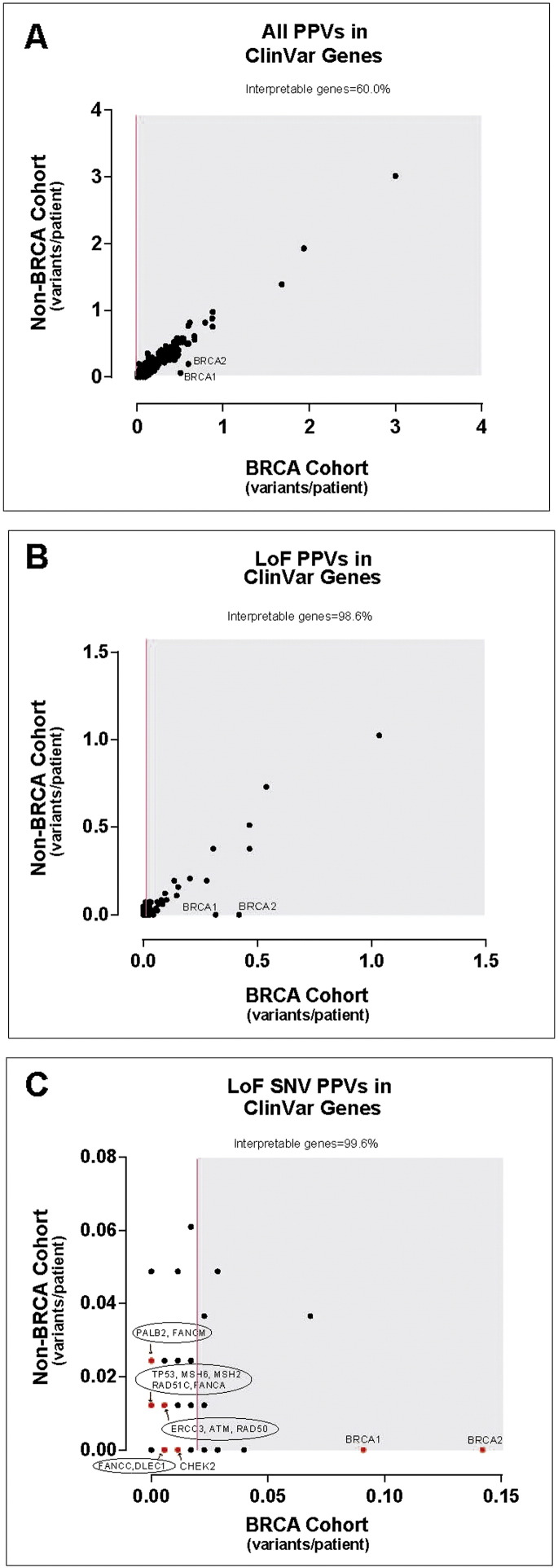
Analysis of PPVs identified by WGS in ClinVar genes. “Gene variance plot” of carrier burden (average number of PPVs per individual) for (A) all PPVs in ClinVar genes, (B) loss-of-function (LoF) PPVs in ClinVar genes or (C) only LoF single nucleotide variant (SNV) PPVs for each gene annotated in the ClinVar database (3209 genes). Genes harboring PPVs in at least 2% of the BRCA1/2 cohort are shown in the shaded region. Those genes not in the shaded region were considered “clinically interpretable” by WGS. One dot can represent several genes. Genes shown in red are those that harbored cancer-risk PPVs in patients, also listed in Table 2, Table 3.

Because several of the non-BRCA-carrier patients were without a genetic diagnosis, we expanded our WGS analysis to include all genes annotated in the ClinVar database (a public collection of 3209 genes associated with human disease; accessed June 2, 2014). However, as the number of genes investigated by WGS increased, so too did the number of PPVs reported to patients, which averaged 111 PPVs per individual. Using the same variance analysis as performed for the 163 disease-genes, only 60.0% of ClinVar genes met our criteria for being clinically interpretable, and this improved to 98.6% when restricting the analysis to LoF PPVs (Fig. 2A and B) and averaged 7.8 LoF PPVs per individual.

To further assess the effect of restricting PPVs to increase specificity, we focused on LoF SNVs, which slightly improved the percent of clinically interpretable genes to 99.6% (Fig. 2C) and averaged only 1.9 LoF SNV PPVs per individual. Only nine of the 82 (11%) non-BRCA individuals had no LoF SNV PPVs in any ClinVar gene. Of the 121 ClinVar genes harboring LoF SNV PPVs, 48 (39.7%) were associated with autosomal dominant diseases (Supplementary Table 17) and 73 (60.3%) were associated with recessive or X-linked diseases (Supplementary Table 18). Of the 131 LoF SNV PPVs, 78 (59.5%) variants in 58 (70.7%) individuals were novel.

Focusing specifically on known cancer-associated genes in the ClinVar database, WGS results were compared to our clinical testing performed as standard of care in the cancer genetics clinic. In addition to confirming mutations diagnosed with standard clinical testing, variant analysis of ClinVar genes identified LoF SNV PPVs in previously unsuspected cancer-associated genes that were not considered as part of standard clinical care (Table 2, Table 3). Of those listed, *ERCC3*, *FANCA* and *FANCM* LoF variants are good candidates for further research as potential cancer-susceptibility mutations as the normal functions of their gene products are in DNA repair pathways. Note that LoF PPVs in ClinVar genes, even if they alter DNA repair pathways, are not necessarily high risk cancer susceptibility mutations. More clinical data is required to quantify risk as was recently done for PALB2 LoF PPVs (Antoniou et al., 2014). This highlights the *potential* for WGS to identify putative cancer risk variants in patients and ultimately increase diagnoses for individuals undergoing genetic testing.

*ERCC3* and *FANCC* LoF variants were also discovered as additional mutations in the BRCA1/2 cohort after analysis of ClinVar gene mutations (Fig. 2C, Table 2). Expanding WGS to ClinVar genes also identified a LoF mutation in the tumor suppressor gene *DLEC1* in a BRCA1-mutant patient (BRCA1.73); the function of the encoded DLEC1 protein is not well defined but has been found to be lost in breast cancer. The DLEC1 acronym stands for “deleted in lung and esophageal cancer” as its expression is lost in these two cancers (and other cancers including prostate and breast) due to somatic mutations or gene silencing (Daigo et al., 1999, Yuan et al., 2003a, Yuan et al., 2003b). We are not aware of earlier reports of *DLEC1* germline mutations associating with familial cancer, illustrating the research value of WGS in the clinic.

## 4 Discussion

The decreasing cost and potential to provide a more comprehensive genetic risk assessment than current targeted methods makes WGS an attractive tool for genetic screening in patients with a family history of disease. For patients in the cancer genetics clinic, WGS may provide new opportunities to better understand their genetic risk for cancer, as well as other (non-cancer) conditions. Therefore, we modeled clinical WGS by performing WGS in patients with and without clinically-confirmed BRCA1/2 mutations. This allowed us to begin to address important questions regarding WGS in the cancer clinic. Particularly, we focused on determining whether a simplified method of interpreting potential clinical implications of individual variants in coding regions of known disease genes would be less burdensome to the patient and clinician and whether WGS provided added value beyond physician-ordered targeted gene or multi-gene panel testing performed as standard of care for our patients.

Initial investigation of the frequency and co-occurrence of PPVs from a limited panel of 163 disease genes in the *BRCA1/2*-carrier cohort indicated that the majority of nonsynonymous PPVs in the cancer genetics clinic will be novel VUSs (not present in the ESP6500 database). Furthermore, our observation of the limited phenotypic effects of previously reported pathogenic missense variants in several genes, as illustrated by the examples of *CREBBP* and *PRSS1,* reinforces how WGS coupled with patient phenotyping will improve the clinical interpretation of genetic diagnoses. That previous estimates of risk for the *CREBBP* and *PRSS1* variants were not predictive in our study cohorts illustrates that clinical-risk prediction for missense variants is frequently in need of additional patient data for confident association with disease in individual patients.

Additional methods based on computational predictions of deleteriousness have been proposed to improve detection of potential disease-causing PPVs, though their clinical use remains unclear. The Combined Annotation-Dependent Depletion (CADD) score represents a summary statistic incorporating multiple predictive methods (Kircher et al., 2014). As expected, a majority of clinically diagnostic LoF PPVs had very high CADD scores; however, only a small proportion of missense PPVs had similarly high CADD scores (Supplemental Fig. 6A). As well, the variability of individual variant CADD scores within a variant class (missense, misstart, etc.) increased as WGS analysis was expanded to all ClinVar genes (Supplemental Fig. 6B). It remains unclear whether CADD scores, or other computational predictions of deleteriousness, will improve clinical interpretation of missense PPVs.

Because of the overwhelming frequency of novel missense PPVs and the difficulty of associating specific PPVs with disease in the published literature, we restricted our analysis to LoF PPVs, which represented a small minority of the PPVs and are predicted to be enriched for pathogenic variants. Limiting reporting to LoF variants in the clinic may assuage some of the fears clinicians feel when contemplating the new era of next-generation sequencing, namely the functional interpretation of VUSs. A similar approach was recently used to investigate the diagnostic rate of WGS in patients with intellectual disability (Gilissen et al., 2014). WGS was performed using the same method reported here (Complete Genomics, Inc.) in 50 patients and focused specifically on *de novo* variants, which required sequencing of parents as well as patients. WGS diagnostic yield and genetic diagnoses were increased if analysis was restricted to LoF sequence variants.

Finally, we expanded WGS analysis to include all human disease-causing genes annotated in the ClinVar database. This potentially represents the largest collection of genes relevant to human health and disease. As expected, expanding WGS analysis increased the number of LoF PPVs; therefore, we further restricted the WGS analysis to LoF SNVs. In total, WGS provided likely genetic cancer risk PPVs in 20.7% [95% CI: 12.6–31.1%] of non-BRCA1/2 clinic patients. A recent report using targeted gene panel testing provided similar cancer risk PPVs in 10.6% [95% CI: 6.1–16.9%] of non-BRCA1/2 patients (Kurian et al., 2014). Therefore, WGS may provide genetic risk predictions for more patients than targeted gene panel testing (p = 0.048), though we expect that significant research efforts in larger patient cohorts will be needed before clinical WGS is widely adopted. We do note that restricting analyses to LoF *SNVs* (as for ClinVar genes) is very conservative and that such an approach would have failed to identify likely cancer risk PPVs in our initial analysis of 163 genes. Comprehensive WGS analysis methods are likely to improve as clinical WGS becomes more widely accepted, and this may require a concerted effort to integrate multi-center WGS results with detailed clinical data from large patient cohorts.

Furthermore, while restricting the analysis to LoF variants for research is feasible, there remains the challenge of false-negatives in the clinical setting. These false-negatives come not only from the conservative interpretation of variants but also from the technical limitations of next-generation sequencing platforms. The use of multiple technologies to supplement genome sequencing, such as microarray analyses or Sanger sequencing, will assist until sequencing technologies and software for variant analysis overcome these challenges.

There are other major challenges involved in translating WGS into the clinic, including questions of mutation penetrance, better understanding of the genes about which we understand little, and how to counsel patients who test negative or positive for an identified familial mutation in one of the less well understood genes (*CHEK2*, *PALB2* or *RAD51C* are good examples of this). Recently we documented that even clinical laboratories are not interpreting the significance of variants consistently (Yorczyk et al., 2014). This increases the urgency for information sharing of variants and their classifications. The more patients we evaluate with detailed and precise family history, the better we will understand the clinical significance of individual genes and, possibly, specific gene mutations. We encourage all clinicians and researchers to use consents that facilitate wide sharing of de-identified data and to submit the results of their studies to public repositories like dbGaP. WGS reported here will be made available through the NCBI dbGaP repository.

Finally, though WGS was performed in these patients, we limited our analysis to the protein-coding exome to facilitate interpretation of nonsynonymous variants. In addition to better clinical interpretation of nonsynonymous (and other) variants, technical improvements will be required to comprehensively detect variants other than SNVs. The overwhelming false-positive rate and lower quality and confidence for detecting insertions and deletions may contribute significantly to the lack of a better diagnosis rate using WGS. Though currently whole-exome sequencing may prove more cost-effective, careful consideration should be paid to understanding the cost–benefit of both methods as technologies continue to improve and sequencing costs decrease, including aspects of sequence uniformity and completeness as well as improvements in variant detection (Biesecker and Green, 2014).

The results from our study highlight the ongoing discussion as to the appropriateness and use of WGS to balance our research goals to improve *future* patient care and risk awareness with the goal of improving our *current* patients' overall health and well-being (Burke and Dimmock, 2014). Proponents of clinical WGS highlight the potential to identify not only genetic risks for the patient's primary diagnosis but also risks for other diseases or conditions that may benefit the patient, such as late-onset diseases or disease risk that may affect reproductive decision-making or family planning. However, contradictory opinions often revolve around the interpretation of sequence results and whether findings unrelated to the current clinical presentation should be returned to patients. While not discussed here, these questions should be addressed for WGS to have the most benefit for current and future patients.

## Role of the Funding Source

This work was partially funded by the 10.13039/100000861Burroughs Wellcome Fund (Clinical Scientist Award ID# 1007448; TSR) and the Peter Bradley Carlson Charitable Trust. TSR holds the Jeanne Ann Plitt Professorship in Breast Cancer Research and the H. Ben and Isabelle T. Decherd Chair in Internal Medicine at UT Southwestern Medical Center, both of which partially funded this research. This research was also supported by the National Center for Advancing Translational Sciences of the National Institutes of Health under award number UL1TR001105. These funders had no role in the study design, data collection, data analysis, interpretation or writing of the report.

## Author Contributions

SBF and JJR interpreted the data and contributed to writing and editing of the manuscript. VM also interpreted the data and contributed to writing and editing of the manuscript. LR, HLH and AET participated in patient consent, sample acquisition and manuscript edits. LD helped interpret the data and isolate DNA from patient blood samples. TSR interpreted the data, participated in patient consent and sample acquisition, wrote and edited the manuscript and led the project.

## Conflict of Interest

The authors have no conflicts of interest.
