# Supplementary material for: Use of Whole Genome Sequencing for Diagnosis and Discovery in the Cancer Genetics Clinic
Source: eBioMedicine. 2014 Dec 3;2(1):74–81. doi: 10.1016/j.ebiom.2014.12.003 (PMC4444225; doi:10.1016/j.ebiom.2014.12.003)
Supplement: Supplementary file 1 — Supplementary material. [file mmc1.pdf]

SUPPLEMENTAL MATERIAL

SIX FIGURES

EIGHTEEN TABLES

SUPPLEMENTAL FIGURE LEGENDS AND FIGURES 1-6

Figure S1. Genotype concordance of WGS from matched lymphoblast and fibroblast samples. Quality scores calculated by Complete Genomics, Inc were used to filter genome-wide variants. Scores ranging from 50-100 were used and compared to no quality score filtering (“0”). Filtering for quality scores greater than 50 had little effect on genotype concordance for SNPs; however, genotype concordance for indels was still poor at a quality score threshold of 100. Average genotype concordance and standard error for all variants (black), SNPs alone (Blue) and indels alone (red) calculated from seven individuals are shown.

Figure S2. Genotype Concordance for genome-wide indels identified by WGS. Average genotype concordance and standard error for indels identified from matched fibroblast and lymphoblast samples from seven individuals was calculated after quality score filtering. No quality score filtering (“0”) is shown with results from filtering quality scores between 50 and 300. Genotype concordance (black) is shown with the proportion of variants remaining after quality filtering (red). Majority of indel variants were excluded to achieve reasonable genotype concordance.

Figure S3. Pedigree of the *BRCA1* 5382insC family with a *CREBBP* p.N1978S mutation. The four generation pedigree of the CREBBP p.Asn1978Ser family with pertinent medical information is shown. Patient identifiers in each generation (generation numbers I-IV to the left of the figure) are located below each symbol. Patient ages at the time of death, if known, are located to the upper right of each symbol. A diagonal line indicates deceased individuals. (CREBBP mt) is positive for the CREBBP p.Asn1978Ser mutation and (CREBBP wt) is wild type CREBBP.

Figure S4. Pedigree of the *BRCA1* 2576delC family with the *PRSS1* p.N29I mutation.

The four generation pedigree of the PRSS1 p.Asn29Ile family with pertinent medical information is shown. Patient identifiers in each generation (generation numbers I-IV to the left of the figure) are located below each symbol. Patient ages at the time of death, if known, are located to the upper right of each symbol. The two patients with an asterisk (*) to the upper left were analyzed with WGS. (PRSS1 mt) is positive for the PRSS1 p.Asn29Ile mutation and (PRSS1 wt) is wild type PRSS1.

Figure S5. Number of PPVs in each of the 163 disease-genes as they relate to coding region length.

A) Number of all PPVs in each gene in the BRCA1/2 cohort. R-square= 0.6082;

B) Number of LoF PPVs in each gene in the BRCA1/2 cohort. R-square= 0.1088;

C) Number of all PPVs in each gene in the non-BRCA cohort. R-square= 0.5217;

D) Number of LoF PPVs in each gene in the non-BRCA cohort. R-square= 0.05069;

R-square is the correlation coefficient.

Figure S6. CADD score distribution of all PPVs identified by WGS in all 258 patients.

The distribution of SNV Phred-scaled CADD scores is shown by variant class for variants identified in the **(A)** selected 163 disease-gene panel and **(B)** all ClinVar genes. As expected, nonsense variant CADD scores were higher than missense variants often defined as variants of unknown significance. LoF PPVs reported as clinically significant in Tables 2 and 3 are red.

SUPPLEMENTAL FIGURES 1-6

Supplemental Figure 1

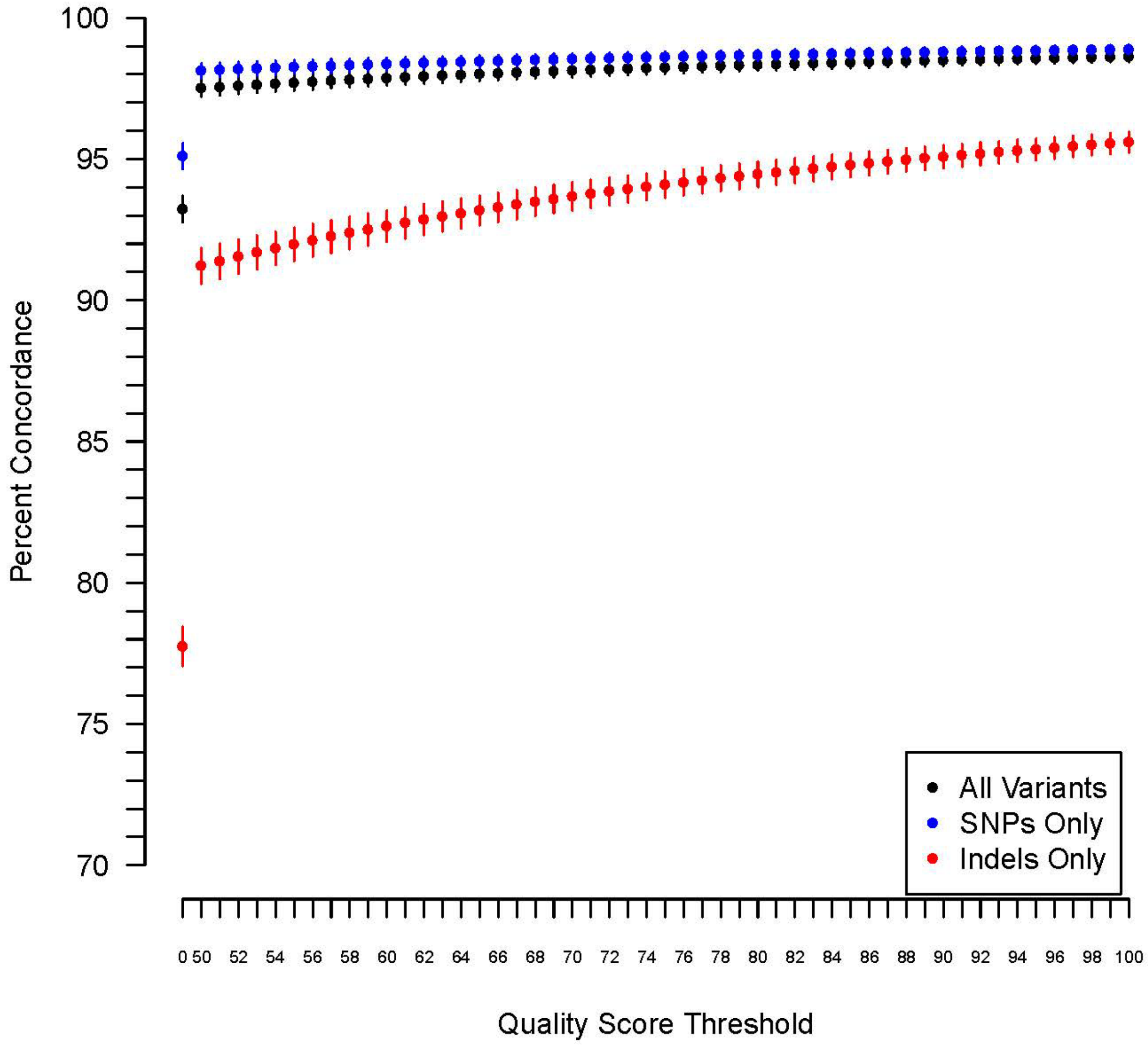

Supplemental Figure 2

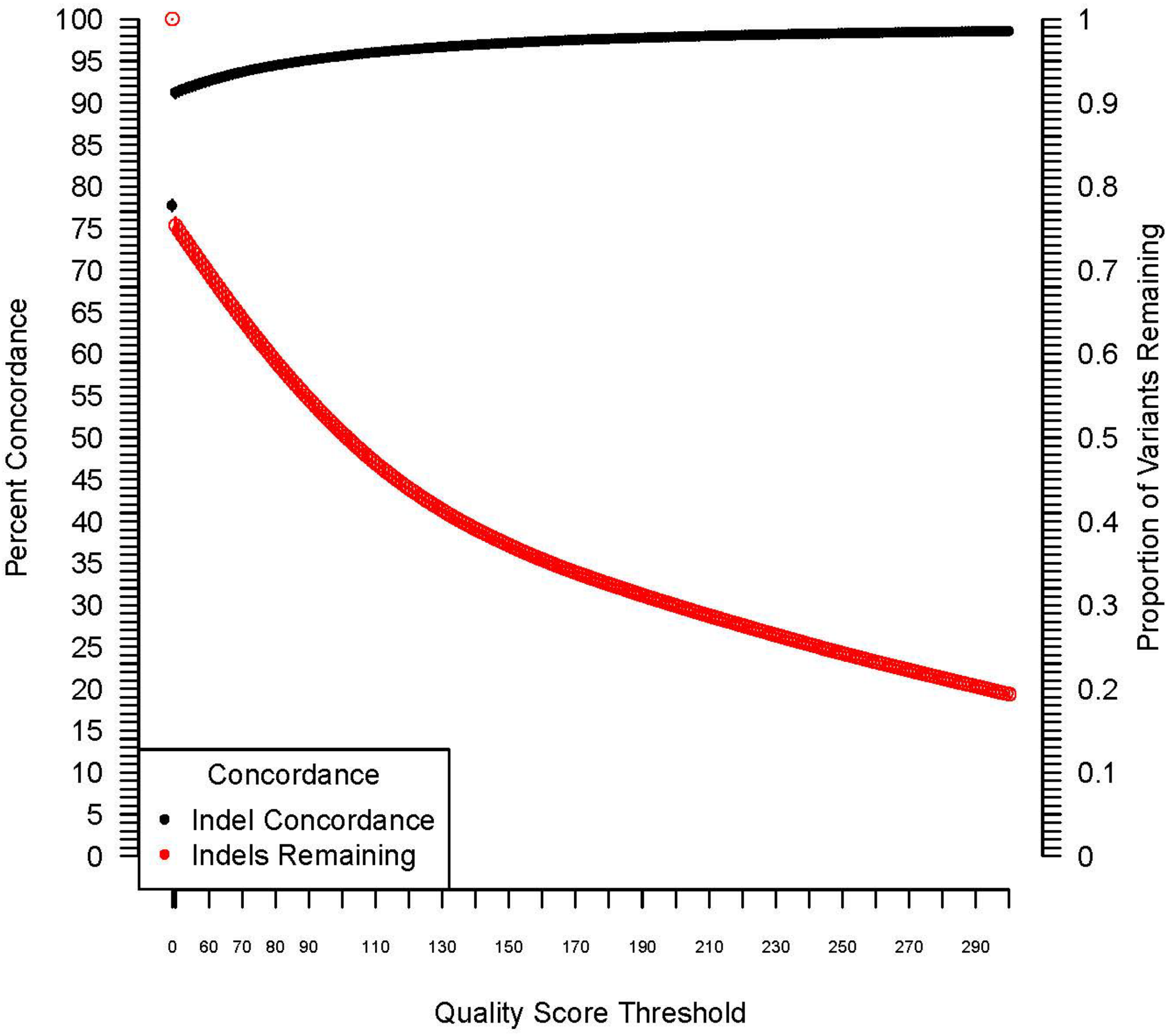

Supplemental Figure 3

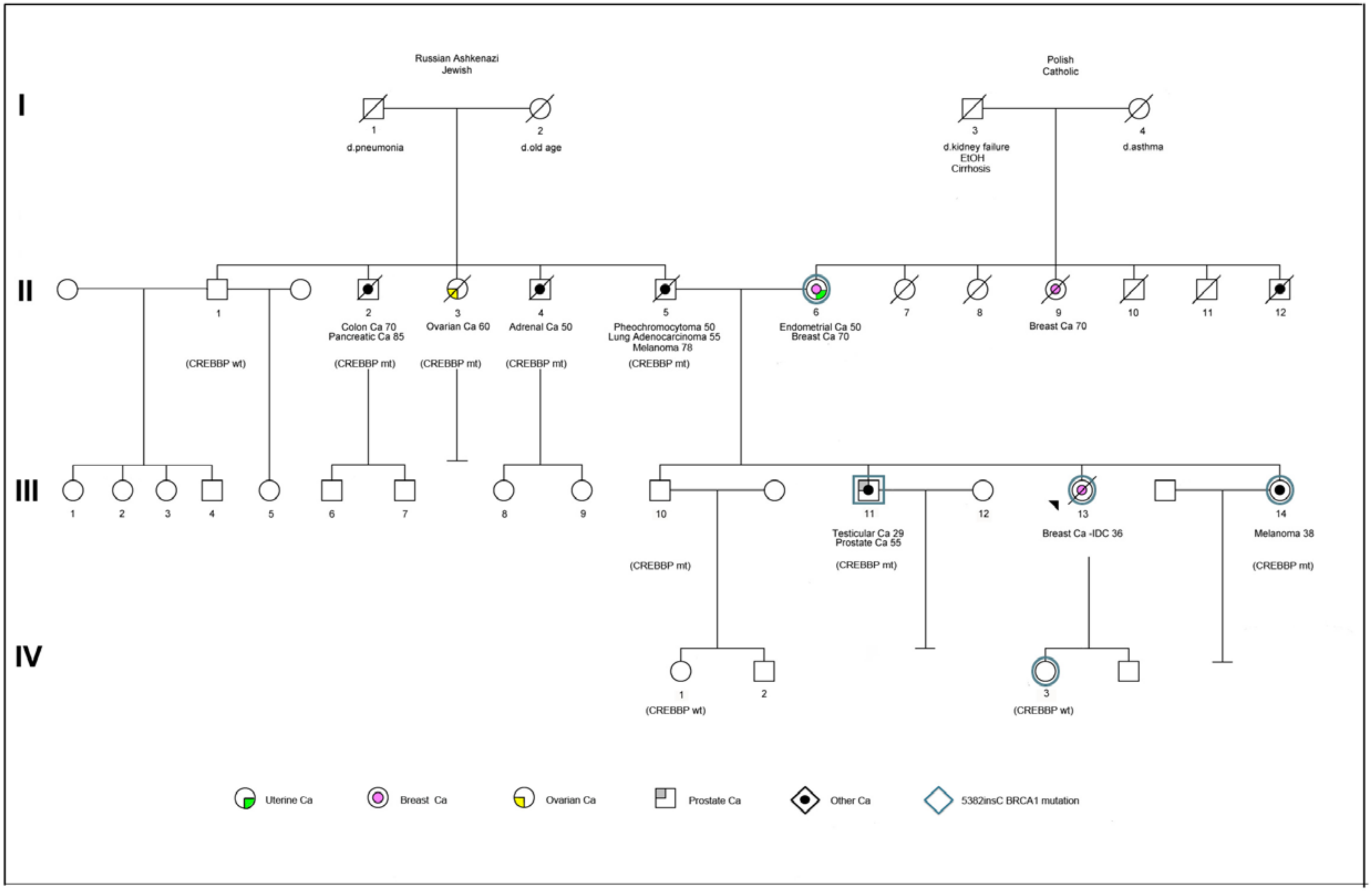

Supplemental Figure 4

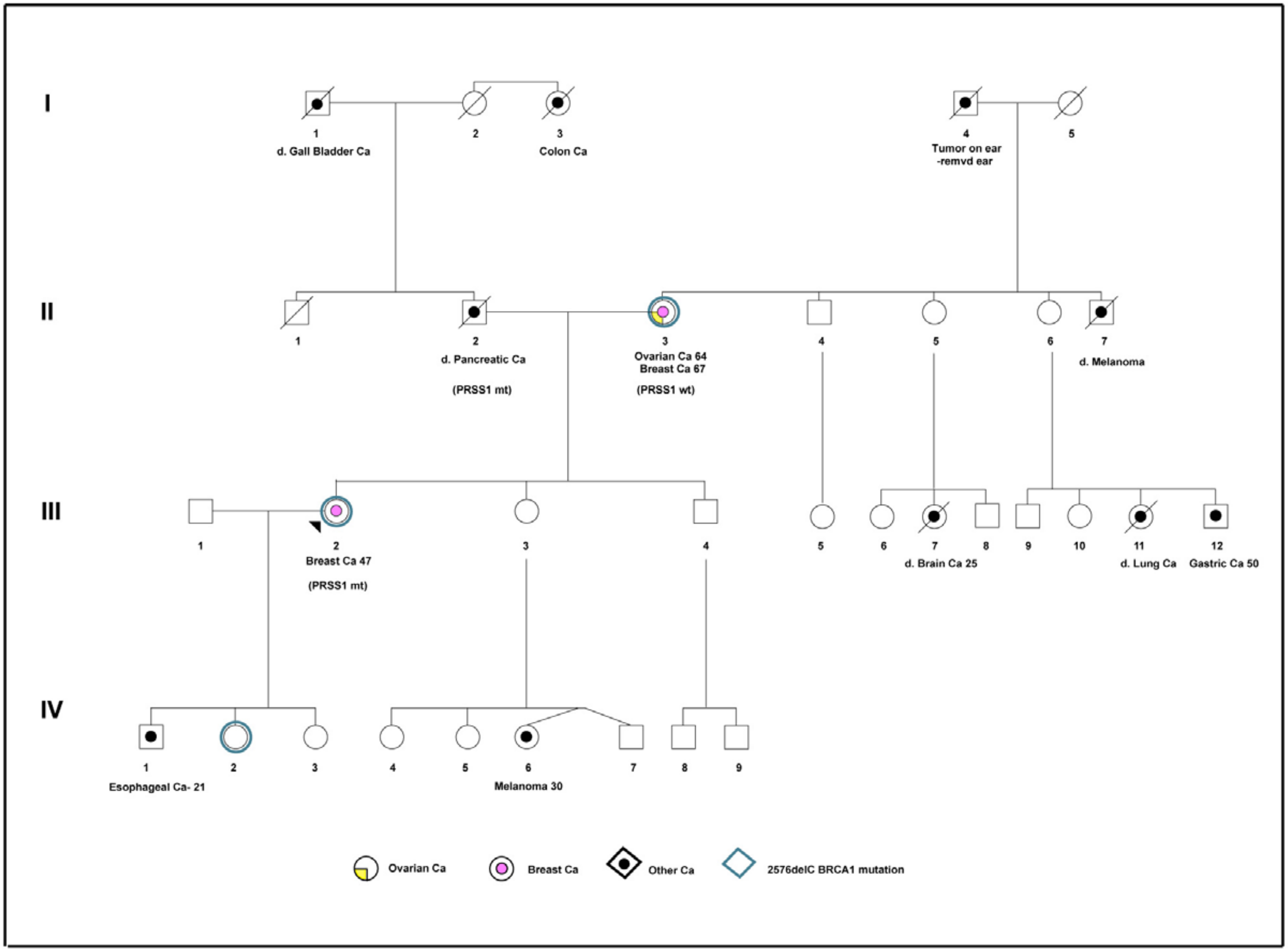

Supplemental Figure 5

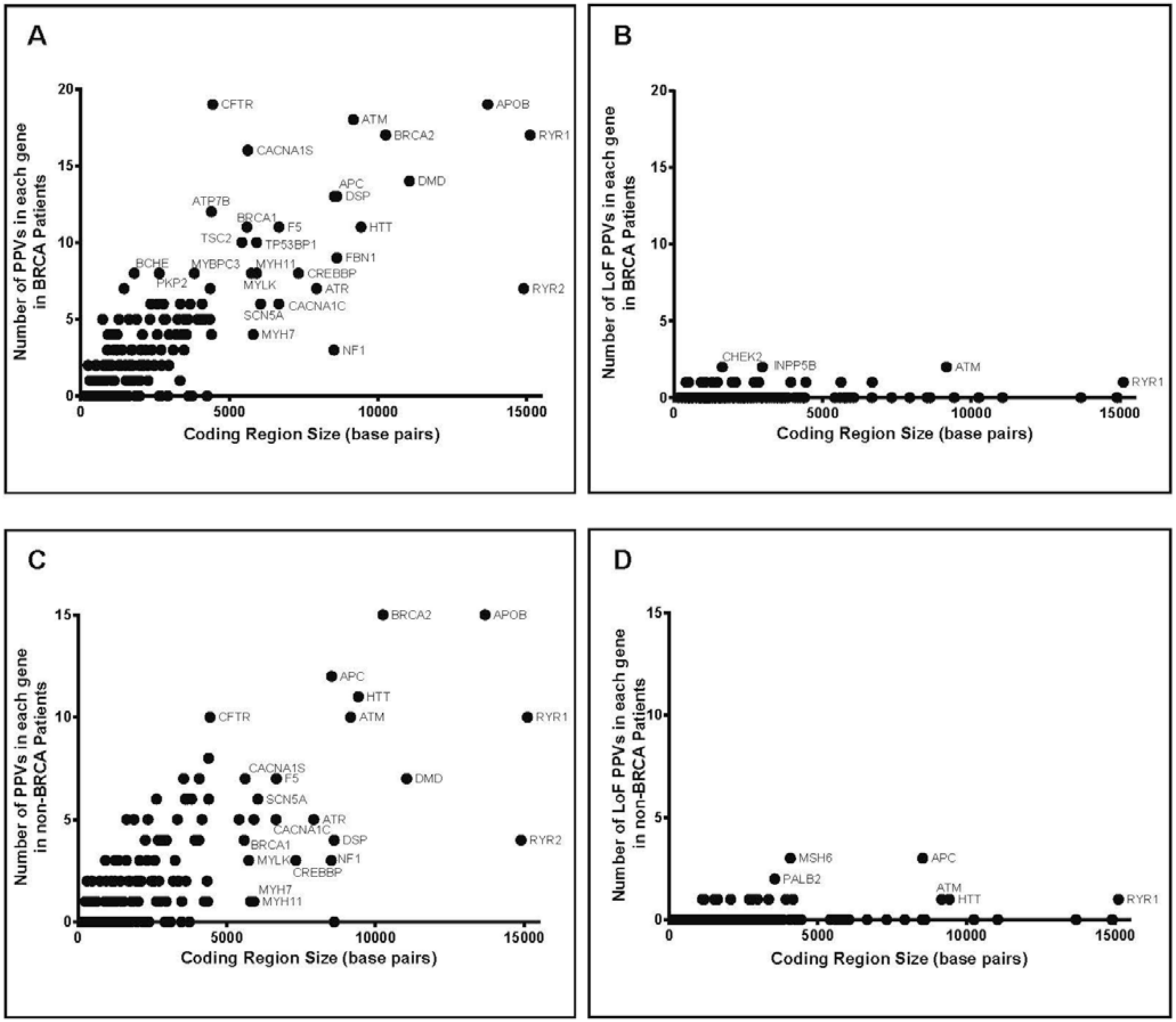

Supplemental Figure 6

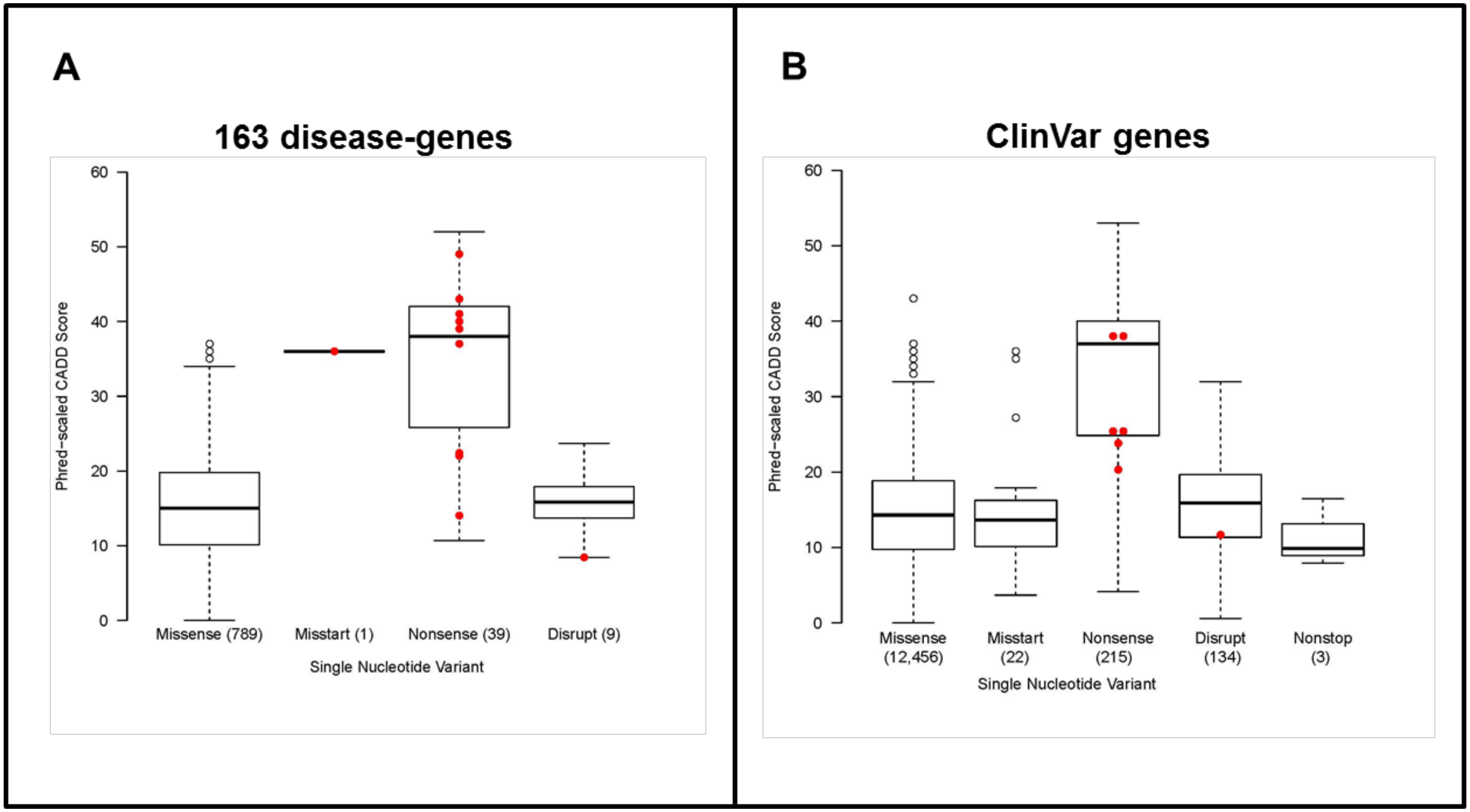

SUPPLEMENTAL TABLES 1-18

Table S1. Summary of Sequencing Statistics

Sample_ID	CGI_Gender	Percent of Genome Fully Sequenced	Percent of Exome Fully Sequenced	Total Sequence (Gb)	Percent of Genome Coverage					Percent of Exome Coverage					Transition/Transversion Ratio		Synonymous SNVs	Nonsynonymous SNVs	Synonymous/ Nonsynonymous Ratio	Missense SNVs	Nonsense SNVs	Nonstop SNVs	Misstart SNVs	Splice SNVs	Frameshift Insertion	Frameshift Deletion	Frameshift Substitution
					At least 5x	At least 10x	At least 20x	At least 30x	At least 40x	At least 5x	At least 10x	At least 20x	At least 30x	At least 40x	Genome	Exome											
**Median of All Samples**		**96.6**	**98**	**158.2125**	**99.3**	**98.3**	**93.05**	**81**	**61.2**	**99.5**	**98.7**	**94.3**	**84**	**66.2**	**2.142**	**3.071**	**10391.75**	**9530.5**	**1.09**	**9430.5**	**73**	**11**	**19**	**72**	**67**	**71**	**20**
BRCA1.1	FEMALE	96.9	98.3	159.164	99.4	98.6	94.4	83.8	64	99.6	98.9	95	85.4	67.5	2.136	3.042	10391	9584	1.08	9482	76	12	14	70	67	68	14
BRCA1.2	FEMALE	96.8	98.2	158.047	99.4	98.6	94.2	83	62.6	99.6	98.9	95.1	85.3	67	2.136	3.048	10437	9663	1.08	9553	76	16	18	69	68	79	15
BRCA1.3	FEMALE	96.9	98.2	161.183	99.4	98.6	94.6	84.6	65.8	99.6	98.9	95.1	85.8	68.4	2.141	3.096	10454	9685	1.08	9583	74	9	19	64	65	67	23
BRCA1.4	FEMALE	96.8	98.2	157.815	99.4	98.5	94	82.9	62.7	99.6	98.9	95	85.3	67.5	2.141	3.042	10398	9450	1.1	9342	79	14	15	70	68	63	23
BRCA1.5	FEMALE	96.9	98.2	161.529	99.4	98.6	94.6	84.6	65.9	99.6	98.8	94.9	85.3	67.6	2.134	3.118	10505	9567	1.1	9447	88	12	20	78	67	66	23
BRCA1.6	FEMALE	96.2	97.3	142.966	99.3	98.2	91.9	76.4	51.3	99.5	98.3	92.3	78.1	54.6	2.145	3.13	10603	9592	1.11	9491	69	11	21	73	52	63	15
BRCA1.7	MALE	96.5	97.7	146.126	99.3	98.1	90.8	75.3	52.4	99.5	98.6	93.5	81.3	59.9	2.146	3.075	10147	9250	1.1	9151	67	13	19	59	61	68	16
BRCA1.8	FEMALE	96.4	97.7	147.853	99.3	98.4	92.7	78.6	54.7	99.5	98.5	93.2	80.5	58.5	2.142	3.075	10229	9491	1.08	9384	78	11	18	65	57	56	17
BRCA1.9	FEMALE	96.3	97.8	146.874	99.3	98.3	92.4	77.5	53.3	99.5	98.6	93.6	80.9	59.2	2.145	3.124	10602	9753	1.09	9637	80	14	22	74	70	71	14
BRCA1.10	FEMALE	96.1	97.6	144.684	99.3	98.2	91.9	76.5	51.9	99.5	98.6	93.3	80.3	58.4	2.147	3.109	12829	11433	1.12	11318	74	15	26	109	57	85	26
BRCA1.11	MALE	96.5	97.6	145.452	99.3	98.1	90.8	75.3	52.3	99.5	98.5	93.2	80.7	58.9	2.142	3.093	10006	9242	1.08	9148	66	9	19	79	62	54	19
BRCA1.12	FEMALE	96.3	97.5	146.365	99.3	98.3	92.6	78.1	53.9	99.5	98.5	93	79.9	57.7	2.144	3.138	12610	11315	1.11	11191	87	15	22	87	71	89	20
BRCA1.13	FEMALE	96.8	98.1	157.261	99.4	98.5	94.1	82.9	62.6	99.6	98.8	94.6	84.4	65.8	2.14	3.037	10460	9466	1.11	9358	78	11	19	76	78	70	18
BRCA1.15	FEMALE	96.8	98.2	159.495	99.4	98.6	94.3	83.7	64	99.6	98.9	95.1	85.6	67.8	2.138	3.071	10554	9791	1.08	9683	76	13	19	75	77	54	22
BRCA1.16	FEMALE	96.6	97.7	153.634	99.2	98.1	92.4	79.9	60	99.4	98.3	93.6	83	65.3	2.145	3.009	10494	9583	1.1	9465	85	12	21	67	54	57	13
BRCA1.17	FEMALE	96.6	97.8	158.236	99.2	98.2	93.1	81.9	63.4	99.4	98.4	93.8	84	67.3	2.148	3.074	10326	9504	1.09	9394	81	9	20	76	61	56	21
BRCA1.18	FEMALE	96.7	98.1	155.266	99.3	98.4	93.5	81.5	60.7	99.6	98.8	94.5	84.1	65.5	2.14	3.086	10368	9433	1.1	9328	72	11	22	66	78	66	23
BRCA1.19	FEMALE	96.6	97.8	152.428	99.2	98.1	92.5	79.6	58.8	99.4	98.4	93.8	83.2	65.2	2.145	3.173	10337	9217	1.12	9103	80	11	23	74	60	60	20
BRCA1.22	FEMALE	96.7	98	154.522	99.3	98.4	93.4	81.1	59.6	99.6	98.8	94.3	83.4	63.9	2.143	3.083	10345	9569	1.08	9464	71	14	20	68	79	76	21
BRCA1.23	FEMALE	96.7	98.3	155.686	99.3	98.4	93.3	81	60	99.7	99	95.5	86.1	68.8	2.142	3.045	10503	9715	1.08	9601	78	14	22	76	59	73	28
BRCA1.24	FEMALE	96.7	97.8	158.207	99.2	98.2	93.2	82	63.7	99.4	98.4	94	84.2	67.6	2.143	3.035	10298	9550	1.08	9453	68	10	19	66	69	67	19
BRCA1.26	FEMALE	96.7	98.3	155.976	99.3	98.4	93.6	81.5	60.3	99.6	98.9	95.1	85	66.6	2.139	3.029	10416	9546	1.09	9438	75	11	22	55	72	73	24
BRCA1.27	FEMALE	96.5	98.1	150.109	99.3	98.4	92.9	79.1	56	99.6	98.9	94.8	83.9	64.2	2.142	3.063	10466	9440	1.11	9341	70	11	18	70	65	75	18
BRCA1.28	FEMALE	96.6	98.1	152.51	99.3	98.4	93.2	80.6	58.7	99.6	98.8	94.5	83.7	64.5	2.138	3.121	10355	9483	1.09	9370	82	11	20	68	57	81	24
BRCA1.29	FEMALE	96.7	98.1	156.617	99.3	98.4	93.5	82	61.9	99.6	98.8	94.5	84.4	66.3	2.137	3.068	10443	9656	1.08	9558	70	11	17	72	79	60	21
BRCA1.30	FEMALE	96.6	97.8	158.001	99.2	98.1	93	81.8	63.5	99.4	98.4	94	84.2	67.7	2.145	3.12	10375	9469	1.1	9381	61	10	17	76	60	55	18
BRCA1.31	FEMALE	96.6	97.9	156.934	99.2	98.1	92.6	80.3	60.9	99.4	98.5	94.2	84.4	67.9	2.145	3.061	10391	9490	1.09	9380	80	12	18	68	63	66	14
BRCA1.32	FEMALE	96.7	97.9	156.398	99.3	98.4	93.5	81.9	61.8	99.5	98.5	94.4	84.8	67.5	2.146	3.078	10481	9545	1.1	9448	67	13	17	69	65	55	18
BRCA1.33	FEMALE	96.3	97.7	145.44	99.3	98.3	92.4	77.5	52.9	99.6	98.6	93.5	80.8	58.7	2.144	3.075	10194	9639	1.06	9529	78	11	21	64	68	61	9
BRCA1.34	FEMALE	96.8	98.3	158.775	99.3	98.5	93.8	82.4	62.3	99.7	99	95.5	86.4	69.5	2.143	3.068	10541	9608	1.1	9507	68	11	22	76	77	88	33
BRCA1.36	FEMALE	96.5	97.9	153.16	99.2	98	92.2	79.5	59.7	99.4	98.5	93.8	83.2	65.7	2.146	3.041	10361	9469	1.09	9375	65	9	20	72	62	62	15
BRCA1.40	FEMALE	96.7	98.2	153.735	99.3	98.4	93.2	80.6	58.7	99.6	98.9	95	84.7	66.1	2.14	3.067	10491	9722	1.08	9617	78	8	19	70	73	75	25
BRCA1.41	FEMALE	96.8	98.3	159.202	99.3	98.5	94	83	63.5	99.6	98.9	95.1	85.8	68.6	2.142	3.019	10310	9438	1.09	9341	70	10	17	80	74	78	27
BRCA1.42	FEMALE	96.7	98.2	156.438	99.3	98.4	93.6	82.1	61.6	99.6	98.9	95	85.3	67.5	2.137	3.026	10506	9656	1.09	9558	68	9	21	65	79	80	14
BRCA1.44	FEMALE	96.8	98.3	160.148	99.3	98.4	93.7	82.6	63.3	99.6	98.9	95.1	85.8	69.1	2.139	3.065	10388	9490	1.09	9382	76	13	19	73	76	68	25
BRCA1.46	FEMALE	96.8	98.2	160.198	99.3	98.4	93.8	82.9	64	99.6	98.8	94.7	84.9	67.6	2.138	3.011	10555	9679	1.09	9582	69	12	16	64	75	81	27
BRCA1.47	FEMALE	96.6	97.9	153.119	99.2	98.1	92.5	79.8	59.3	99.4	98.5	94.1	83.8	66.2	2.143	3.069	10305	9584	1.08	9477	75	11	21	73	69	59	16
BRCA1.48	FEMALE	96.1	97.8	142.914	99.3	98.1	91.2	74.5	49.7	99.6	98.8	94.1	81.5	60.7	2.149	3.081	10496	9515	1.1	9418	71	7	19	83	61	77	23
BRCA1.49	FEMALE	96.2	97.9	143.558	99.3	98.3	92	76.4	51.7	99.6	98.8	94.2	82.1	61	2.147	3.082	10282	9394	1.09	9299	67	12	16	80	65	75	13
BRCA1.50	FEMALE	96.9	98.2	162.029	99.4	98.6	94.4	84.4	65.8	99.6	98.9	95.1	85.9	69	2.136	3.048	10316	9580	1.08	9483	71	6	20	80	65	72	26
BRCA1.52	FEMALE	96.8	98.2	161.1	99.4	98.5	94.2	83.7	64.9	99.6	98.9	95	85.7	68.7	2.14	3.062	10279	9505	1.08	9403	68	15	19	70	74	75	25
BRCA1.53	FEMALE	96.5	97.8	158.729	99.2	98.1	92.6	81	63	99.4	98.4	93.8	83.8	67.6	2.143	3.07	10526	9711	1.08	9602	76	12	21	70	62	56	19
BRCA1.55	FEMALE	96.6	97.6	153.3	99.2	98.1	92.6	80.4	60.7	99.4	98.2	93.1	82	63.7	2.14	3.077	10329	9387	1.1	9291	68	10	18	72	68	65	28
BRCA1.56	FEMALE	96.7	97.8	155.796	99.3	98.2	93.2	81.9	62.9	99.4	98.4	93.8	83.7	66.5	2.143	3.101	10398	9533	1.09	9434	72	7	20	75	54	46	19
BRCA1.57	MALE	96.8	98.3	150.616	99.3	98.1	91.3	76.7	55.1	99.6	98.9	95	84.6	65.8	2.143	3.059	10358	9537	1.09	9422	81	13	21	65	70	73	18
BRCA1.58	FEMALE	96.6	97.8	154.85	99.2	98.1	92.7	80.7	61.5	99.4	98.4	93.8	83.4	66.2	2.14	3.07	10365	9419	1.1	9307	80	11	21	68	55	65	15
BRCA1.59	FEMALE	96.6	98.1	152.137	99.3	98.3	92.9	79.8	57.5	99.6	98.8	94.6	83.7	64.4	2.14	3.086	10459	9552	1.09	9454	70	10	18	62	72	67	19
BRCA1.60	FEMALE	96.8	98	157.952	99.3	98.3	93.4	82.2	62.9	99.4	98.5	94.2	84.5	67.4	2.139	3.037	10499	9719	1.08	9629	64	10	16	66	78	70	15
BRCA1.61	FEMALE	96.6	98.1	151.75	99.4	98.5	93.6	80.6	57.7	99.6	98.9	95	84.5	64.8	2.143	3.091	10261	9337	1.1	9246	63	9	19	64	67	81	16
BRCA1.62	FEMALE	96.8	98.1	158.45	99.4	98.5	94.2	83.4	63.7	99.6	98.8	94.7	84.8	66.6	2.136	3.084	10544	9660	1.09	9551	78	13	18	68	68	84	19
BRCA1.63	FEMALE	96.8	98.1	158.835	99.4	98.5	94.1	83.3	63.7	99.6	98.7	94.2	83.8	65.1	2.136	3.02	10497	9670	1.09	9561	77	11	21	76	71	70	16
BRCA1.64	FEMALE	96.6	98.1	155.026	99.3	98.3	93.1	80.6	59.3	99.6	98.9	94.9	84.9	67	2.145	3.118	10469	9664	1.08	9565	65	11	23	74	67	80	21
BRCA1.65	MALE	96.9	98.1	153.948	99.3	98.1	91.8	78.3	58.2	99.6	98.8	94.7	84.7	66.7	2.14	3.065	10309	9545	1.08	9450	67	12	16	70	67	69	21
BRCA1.66	FEMALE	96.8	98.2	157.666	99.3	98.5	93.9	82.5	62.2	99.6	98.9	95.2	85.7	68.1	2.136	3.055	10396	9516	1.09	9403	80	13	20	79	64	85	28
BRCA1.67	FEMALE	96.6	97.9	156.606	99.2	98.1	92.8	81	62	99.4	98.4	94	84	67.1	2.142	3.094	10418	9435	1.1	9340	63	12	20	67	66	62	17
BRCA1.68	FEMALE	96.5	97.7	156.779	99.2	98	92.3	80.1	61.4	99.4	98.3	93.5	83	66.2	2.144	3.074	10269	9373	1.1	9281	64	9	19	70	51	49	13
BRCA1.69	FEMALE	96.6	98.1	152.087	99.4	98.4	93.4	80.5	58.2	99.6	98.9	94.9	84.5	65.4	2.144	3.088	10357	9493	1.09	9394	68	10	21	72	62	78	21
BRCA1.71	FEMALE	96.3	97.8	141.112	99.3	98.3	91.9	75.9	50.1	99.5	98.6	93.1	79.3	55.9	2.147	3.073	10243	9460	1.08	9355	72	15	18	72	60	90	15
BRCA1.72	FEMALE	96.5	97.8	154.543	99.2	98.1	92.3	79.6	59.7	99.4	98.5	94.1	84	67.1	2.147	3.075	10355	9396	1.1	9300	67	9	20	67	62	63	12
BRCA1.73	FEMALE	96.6	97.9	155.794	99.2	98.1	92.7	80.7	61.4	99.4	98.4	93.9	83.8	66.8	2.145	3.083	10354	9608	1.08	9500	76	14	18	75	56	59	23
BRCA1.74	FEMALE	96.7	97.9	158.391	99.3	98.2	93.3	82.1	63.6	99.4	98.4	94	84.4	67.8	2.145	3.135	10347	9464	1.09	9351	79	12	22	70	62	59	19
BRCA1.75	FEMALE	96.2	98	145.81	99.3	98.3	92.1	76.7	52.6	99.6	98.9	94.5	82.8	62.5	2.147	3.079	10245	9469	1.08	9372	68	9	20	65	60	80	20
BRCA1.77	FEMALE	96.7	98	155.042	99.3	98.4	93.6	81.8	61	99.6	98.8	94.3	83.7	64.6	2.137	3.037	10220	9496	1.08	9394	77	9	16	75	76	79	24
BRCA1.78	FEMALE	96.5	97.7	155.916	99.2	98.1	92.4	80.3	61.2	99.4	98.3	93.6	83.2	66.2	2.149	3.079	10315	9428	1.09	9326	68	14	20	74	60	63	21
BRCA1.80	FEMALE	96.6	97.9	157.425	99.2	98.1	92.8	81	62.2	99.4	98.5	94.3	84.6	68.3	2.14	3.029	10584	9758	1.08	9644	84	11	19	71	64	74	22
BRCA1.81	FEMALE	95.9	97.8	142.483	99.3	98	90.9	74.1	49.8	99.6	98.8	93.9	81.4	60.9	2.152	3.098	10343	9561	1.08	9463	64	14	20	78	60	71	16
BRCA1.83	FEMALE	96.3	97.6	147.634	99.2	97.9	91.4	77.3	55.4	99.4	98.2	92.6	80.2	60.1	2.145	3.132	10223	9358	1.09	9256	68	10	24	70	52	58	23
BRCA1.84	FEMALE	96.1	97.9	145.977	99.3	98.1	91.4	75.5	51.8	99.6	98.8	94.2	82.4	62.6	2.147	3.108	10267	9519	1.08	9419	70	10	20	77	61	68	17
BRCA1.85	FEMALE	96.5	98.1	178.385	99.4	98.6	94.6	85.2	69.1	99.7	99.1	96.3	89.4	76.8	2.143	3.099	10472	9506	1.1	9398	85	8	15	77	62	94	11
BRCA1.86	FEMALE	96.8	98.2	159.411	99.3	98.5	93.9	82.8	63.1	99.6	98.9	94.9	85.2	67.6	2.136	3.082	10529	9557	1.1	9448	75	12	22	63	62	72	22
BRCA1.87	FEMALE	96.1	97.9	144.671	99.3	98.2	91.8	75.8	51.4	99.6	98.8	94.2	82.1	61.4	2.144	3.059	10172	9507	1.07	9404	76	10	17	72	62	85	16
BRCA1.90	FEMALE	96	97.6	140.569	99.3	98.1	91	73.7	48.2	99.6	98.7	93.4	79.8	57.7	2.146	3.077	10162	9320	1.09	9219	71	12	18	79	50	76	20
BRCA1.91	FEMALE	96	97.9	143.124	99.3	98	90.8	73.5	48.4	99.6	98.8	93.8	80.6	59	2.149	3.062	10395	9568	1.09	9469	68	13	18	68	61	70	23
BRCA1.93	FEMALE	96.3	98	147.802	99.4	98.4	92.6	77.9	54.4	99.6	98.9	94.6	83.3	63.3	2.146	3.094	10261	9529	1.08	9431	69	10	19	70	57	55	19
BRCA1.94	FEMALE	96.2	97.8	147.64	99.4	98.3	92.4	77.4	53.5	99.6	98.8	94.4	82.6	62	2.148	3.067	12060	10856	1.11	10752	71	10	23	73	67	74	20
BRCA1.96	FEMALE	96.3	97.9	145.978	99.3	98.3	92.3	77.1	52.7	99.6	98.8	94.2	82.3	61.3	2.144	3.079	10315	9539	1.08	9441	68	8	22	66	62	83	14
BRCA1.97	FEMALE	96.5	98	152.617	99.4	98.4	93.1	79.6	57.2	99.6	98.9	94.6	83.4	63.7	2.145	3.061	10428	9490	1.1	9394	63	11	22	85	71	87	28
BRCA1.98	FEMALE	96.8	98	159.886	99.3	98.3	93.4	82.4	64.2	99.5	98.6	94.4	84.9	68.4	2.142	3.085	10398	9541	1.09	9440	73	9	19	75	57	67	24
BRCA1.99	FEMALE	96.6	98.1	155.048	99.4	98.5	93.7	81.3	59.7	99.6	98.9	94.8	84.3	65.3	2.143	3.085	10332	9618	1.07	9522	66	13	17	72	71	91	17
BRCA1.100	FEMALE	96.5	98.2	153.75	99.3	98.4	93.2	80.4	58.6	99.6	99	95.1	85.1	67	2.142	3.058	10507	9716	1.08	9600	83	12	21	68	66	81	23
BRCA1.102	FEMALE	96.5	98.3	151.176	99.3	98.2	92.5	78.7	56.3	99.7	99	95.2	85	66.9	2.147	3.112	10310	9416	1.09	9324	65	10	17	69	61	61	12
BRCA1.104	FEMALE	96.6	97.8	156.904	99.2	98.1	92.7	80.9	62	99.4	98.3	93.6	83	65.7	2.142	3.082	10155	9279	1.09	9179	70	10	20	69	73	57	23
BRCA1.105	FEMALE	96.7	97.7	156.58	99.3	98.2	93.1	81.7	62.8	99.4	98.3	93.7	83.5	66.3	2.142	3.098	10238	9600	1.07	9508	63	12	17	85	65	64	9
BRCA1.106	FEMALE	96.3	98	144.549	99.3	98.3	92	76.5	51.8	99.6	98.8	94.4	82.4	61.6	2.144	3.087	12715	11404	1.11	11285	79	16	24	78	69	91	17
BRCA1.107	FEMALE	96.5	98.1	148.473	99.3	98.3	92.6	78.1	54.2	99.6	98.9	94.8	83.6	63.7	2.146	3.058	10320	9446	1.09	9340	76	11	19	75	67	65	21
BRCA1.108	FEMALE	95.9	97.9	170.139	99	97.2	89.1	75.6	59.2	99.6	98.7	94.2	85.1	73	2.144	3.009	10566	9767	1.08	9661	79	9	18	65	71	64	26
BRCA1.111	FEMALE	96.3	97.9	159.958	99.2	98	91.9	78.4	58.4	99.5	98.7	94	82.7	64.6	2.144	3.07	10580	9431	1.12	9333	67	11	20	83	59	85	22
BRCA1.112	FEMALE	96.5	97.7	164.722	99.3	98.2	92.7	80.2	60.9	99.5	98.4	93.2	81.3	62.8	2.143	3.107	12623	11004	1.15	10892	79	13	20	83	64	83	21

Table S1 continued

Sample_ID	CGI_Gender	Percent of Genome Fully Sequenced	Percent of Exome Fully Sequenced	Total Sequence (Gb)	Percent of Genome Coverage					Percent of Exome Coverage					Transition/Transversion Ratio		Synonymous SNVs	Nonsynonymous SNVs	Synonymous/ Nonsynonymous Ratio	Missense SNVs	Nonsense SNVs	Nonstop SNVs	Misstart SNVs	Splice SNVs	Frameshift Insertion	Frameshift Deletion	Frameshift Substitution
					At least 5x	At least 10x	At least 20x	At least 30x	At least 40x	At least 5x	At least 10x	At least 20x	At least 30x	At least 40x	Genome	Exome											
**Median of All Samples**		**96.7**	**98**	**159.267**	**99.3**	**98.4**	**93.1**	**81.3**	**61.5**	**99.5**	**98.7**	**94.3**	**84**	**66.2**	**2.142**	**3.067**	**10394**	**9529**	**1.09**	**9428**	**73**	**11**	**19**	**73**	**67**	**72**	**20**
BRCA2.1	MALE	96.9	98.1	156.683	99.3	98.4	92.8	80.4	61.1	99.6	98.7	94.5	84.5	66.5	2.138	3.052	10484	9482	1.11	9380	74	11	17	73	68	61	24
BRCA2.2	FEMALE	96.7	98.3	159.414	99.4	98.5	93.9	82.7	63.2	99.6	99	95.4	86.2	69.3	2.141	3.053	10292	9480	1.09	9368	83	10	19	64	76	71	18
BRCA2.3	FEMALE	96.7	98.1	156.822	99.4	98.5	94.1	83.1	63.1	99.6	98.8	94.8	85	67	2.141	3.054	10429	9661	1.08	9564	72	11	14	80	68	62	16
BRCA2.4	FEMALE	96.7	98.2	155.886	99.4	98.5	93.8	82.2	61.4	99.6	99	95.3	85.9	68.3	2.143	3.088	10340	9461	1.09	9351	80	9	21	61	66	73	15
BRCA2.5	FEMALE	96.7	98.3	156.227	99.4	98.5	93.7	81.9	61.2	99.6	99	95.3	85.8	68.1	2.143	3.015	10508	9697	1.08	9589	79	10	19	70	70	83	17
BRCA2.6	FEMALE	96.7	98.2	156.918	99.4	98.5	94.1	83.1	63.2	99.6	98.9	95.2	85.9	68.4	2.144	3.092	11941	10723	1.11	10618	73	14	18	73	68	68	16
BRCA2.7	FEMALE	96.8	98.1	158.226	99.4	98.6	94.3	83.6	63.7	99.6	98.8	94.6	84.5	66.2	2.145	3.088	10346	9565	1.08	9468	71	10	16	85	68	78	23
BRCA2.9	FEMALE	96.7	98.2	156.434	99.4	98.5	93.9	82.3	61.6	99.6	98.9	95	85.1	66.8	2.142	3.052	10390	9450	1.1	9345	77	10	18	70	64	69	21
BRCA2.11	FEMALE	96.7	98.3	155.096	99.4	98.5	93.8	82	61	99.6	98.9	95.2	85.6	67.6	2.144	3.048	10345	9592	1.08	9485	74	14	19	75	71	61	26
BRCA2.12	FEMALE	96.8	98.2	158.378	99.4	98.5	93.9	82.8	63.2	99.6	98.9	95	85.3	67.7	2.138	3.024	10344	9478	1.09	9385	64	13	16	76	76	70	17
BRCA2.13	FEMALE	96.8	98.1	158.198	99.4	98.5	94.2	83.3	63.4	99.6	98.9	95.1	85.5	67.7	2.14	3.029	10379	9433	1.1	9317	81	15	20	80	70	70	21
BRCA2.14	FEMALE	96.9	98.1	158.532	99.4	98.6	94.3	83.7	64.1	99.6	98.8	94.7	84.7	66.4	2.138	3.08	10437	9552	1.09	9439	77	13	23	73	70	62	18
BRCA2.15	MALE	97	98.2	158.185	99.3	98.4	92.9	80.6	61.5	99.6	98.9	95.2	85.9	68.5	2.137	3.067	10398	9491	1.1	9392	65	14	20	74	68	74	14
BRCA2.16	FEMALE	96.7	98.2	159.542	99.3	98.5	93.8	82.6	62.9	99.6	98.9	95.3	85.9	68.8	2.14	3.089	10568	9545	1.11	9446	66	12	21	76	71	81	22
BRCA2.17	FEMALE	96.8	98.3	159.482	99.4	98.5	94.1	82.9	62.9	99.6	99	95.4	86.1	68.8	2.141	3.088	10404	9678	1.08	9567	78	13	20	65	71	74	27
BRCA2.18	FEMALE	96.6	98.1	154.587	99.3	98.4	93.4	81.2	59.9	99.6	98.9	94.8	84.4	65.3	2.14	3.063	10504	9593	1.09	9471	85	15	22	72	66	74	18
BRCA2.19	FEMALE	96.8	98.1	158.908	99.4	98.6	94.3	83.5	63.8	99.6	98.9	94.9	85.1	67	2.138	3.056	10317	9551	1.08	9452	72	10	17	63	73	83	22
BRCA2.21	FEMALE	96.8	98.2	159.693	99.4	98.6	94.3	83.7	64.1	99.6	98.9	95.2	85.7	68	2.14	3.076	10466	9629	1.09	9524	72	13	20	69	64	77	24
BRCA2.22	FEMALE	96.8	98.1	159.249	99.4	98.5	94.2	83.5	63.8	99.6	98.8	94.8	85	67	2.139	3.093	10305	9436	1.09	9333	75	12	16	68	64	72	22
BRCA2.23	FEMALE	96.7	98	155.275	99.3	98.4	93.6	81.8	61.2	99.5	98.7	94.4	83.9	65.4	2.14	3.002	10449	9490	1.1	9383	79	10	18	77	76	60	24
BRCA2.24	FEMALE	96.7	98.2	158.684	99.4	98.5	94	82.8	62.6	99.6	99	95.4	86.3	68.9	2.143	3.06	12178	10857	1.12	10743	76	17	21	88	65	73	16
BRCA2.25	FEMALE	96.7	98.1	157.898	99.4	98.5	94	83	63.3	99.6	98.8	94.6	84.8	67	2.142	3.076	10304	9464	1.09	9371	66	9	18	72	73	62	25
BRCA2.26	FEMALE	96.8	98.3	160.529	99.4	98.6	94.4	83.9	64.6	99.6	99	95.6	86.9	70.3	2.14	3.045	10445	9647	1.08	9542	76	10	19	74	65	78	23
BRCA2.27	FEMALE	96.7	97.9	156.329	99.4	98.5	94	82.8	62.5	99.5	98.7	94.2	83.8	64.9	2.142	3.112	10386	9465	1.1	9348	84	12	21	76	73	54	17
BRCA2.28	FEMALE	96.8	98	157.925	99.4	98.6	94.2	83.4	63.6	99.6	98.7	94.6	84.8	66.8	2.14	3.058	10328	9529	1.08	9418	79	11	21	76	69	58	25
BRCA2.29	FEMALE	96.6	98.1	153.481	99.3	98.4	93.4	81.2	60.2	99.6	98.8	94.6	84.5	66.2	2.144	3.028	10397	9448	1.1	9343	77	10	18	78	65	73	18
BRCA2.31	FEMALE	96.8	98	160.358	99.4	98.6	94.5	84.3	65.4	99.6	98.7	94.6	84.9	67.3	2.139	3.082	10375	9379	1.11	9277	66	15	21	65	68	63	22
BRCA2.34	FEMALE	96.8	98.2	158.749	99.4	98.5	94.1	83.2	63.9	99.6	98.8	94.7	84.8	66.9	2.137	3.09	10444	9626	1.08	9521	76	11	18	70	57	89	19
BRCA2.35	FEMALE	96.7	98.1	157.83	99.4	98.5	93.9	82.8	62.8	99.6	98.9	95	85.6	68.4	2.142	3.026	10470	9574	1.09	9465	78	8	23	84	77	68	18
BRCA2.36	FEMALE	96.2	97.6	141.098	99.3	98.2	91.6	75.2	49.5	99.5	98.5	92.6	78.4	54.8	2.147	3.074	10197	9343	1.09	9250	66	11	16	75	68	62	19
BRCA2.37	FEMALE	96.7	98.2	157.069	99.4	98.5	93.9	82.6	62.5	99.6	98.9	95	85.4	67.7	2.14	3.046	10303	9495	1.09	9390	74	9	22	66	65	77	27
BRCA2.38	FEMALE	96.7	98.2	156.646	99.4	98.5	93.8	82.1	61.6	99.6	98.9	95.2	85.7	67.9	2.144	3.063	10474	9577	1.09	9464	78	13	22	71	66	67	10
BRCA2.39	FEMALE	96.8	98.2	158.982	99.4	98.5	94.3	83.6	64.2	99.6	98.8	94.9	85.4	67.7	2.144	3.04	10283	9495	1.08	9391	74	12	18	66	67	67	15
BRCA2.40	FEMALE	96.7	98.1	153.088	99.3	98.4	93.6	81.5	60.3	99.6	98.8	94.5	84	64.9	2.142	3.058	10362	9689	1.07	9588	75	12	14	68	79	66	18
BRCA2.41	FEMALE	96.7	98.1	155.852	99.4	98.5	93.8	82.3	61.7	99.6	98.8	94.7	84.7	66.2	2.141	3.11	10278	9500	1.08	9394	79	9	18	66	63	68	16
BRCA2.42	MALE	96.9	98.2	155.653	99.3	98.2	92.2	79.2	59.3	99.6	98.9	95.2	85.9	68.7	2.142	3.071	10391	9630	1.08	9519	80	10	21	74	66	64	22
BRCA2.43	FEMALE	96.8	98.4	160.205	99.4	98.5	94.2	83.4	64.2	99.7	99.1	96.1	88	72.5	2.144	3.063	10352	9465	1.09	9365	68	10	22	72	63	66	12
BRCA2.44	FEMALE	96.8	98.2	159.832	99.4	98.5	94.1	83.2	64	99.6	98.9	94.9	85.3	67.9	2.139	3.126	10500	9488	1.11	9393	70	9	16	78	61	74	20
BRCA2.48	FEMALE	96.7	98.2	156.576	99.3	98.5	93.7	82.2	62	99.6	98.9	95.3	86.1	68.9	2.145	3.064	10565	9664	1.09	9558	85	8	13	68	66	81	19
BRCA2.49	FEMALE	96.7	98.1	158.049	99.4	98.5	94	83	63.1	99.6	98.8	94.8	85.2	67.6	2.139	3.081	10520	9631	1.09	9525	75	11	20	80	62	73	11
BRCA2.50	FEMALE	96.8	98.2	157.512	99.4	98.5	94.2	83.3	63.7	99.6	98.8	95	85.6	68.1	2.143	3.072	10338	9585	1.08	9488	67	10	20	72	66	78	18
BRCA2.51	FEMALE	96.8	98	157.003	99.4	98.6	94.2	83.3	63.4	99.5	98.7	94.3	83.9	65.2	2.141	3.057	10422	9676	1.08	9570	78	11	17	79	70	67	17
BRCA2.52	FEMALE	96.8	98.2	160.348	99.4	98.6	94.3	83.7	64.4	99.6	98.9	95.3	86	68.8	2.144	3.056	10395	9732	1.07	9630	72	9	21	71	78	65	28
BRCA2.53	FEMALE	96.8	98.1	158.053	99.4	98.5	94.1	82.9	62.8	99.6	98.9	94.9	85.1	67	2.14	3.07	10438	9484	1.1	9378	75	11	20	65	70	84	24
BRCA2.54	FEMALE	96.7	98.1	158.912	99.4	98.5	94.2	83.4	63.8	99.6	98.8	94.9	85.3	67.8	2.142	3.027	10419	9471	1.1	9379	65	13	14	69	62	62	27
BRCA2.55	FEMALE	96.7	98.2	155.946	99.4	98.5	93.9	82.3	61.5	99.6	98.9	94.9	84.9	66.5	2.139	3.087	10373	9473	1.1	9374	68	10	21	70	74	71	17
BRCA2.56	FEMALE	96.7	98	156.068	99.4	98.5	94.1	82.8	62.5	99.5	98.6	94	83.2	63.9	2.142	3.067	10199	9437	1.08	9329	79	11	18	71	59	78	18
BRCA2.58	FEMALE	96.7	98	158.341	99.4	98.6	94.2	83.4	63.6	99.6	98.7	94.5	84.6	66.4	2.142	3.129	12496	11264	1.11	11143	80	14	27	88	75	81	18
BRCA2.59	FEMALE	96.7	98.1	153.531	99.4	98.5	93.5	81.2	59.8	99.6	98.8	94.6	84.1	65.1	2.142	3.052	10384	9490	1.09	9396	67	12	15	68	72	69	19
BRCA2.61	FEMALE	96.6	97.9	150.984	99.4	98.4	93.4	80.7	58.4	99.5	98.7	93.9	82.3	61.6	2.143	3.065	10336	9520	1.09	9423	71	10	16	68	71	59	22
BRCA2.63	FEMALE	96.7	98.2	156.782	99.3	98.4	93.6	81.8	61	99.6	98.9	95.2	85.7	68.1	2.147	3.03	10206	9575	1.07	9478	67	13	17	79	67	82	23
BRCA2.65	FEMALE	96.7	98.2	158.33	99.4	98.5	94	82.7	62.6	99.6	98.9	95.2	85.8	68.6	2.141	3.047	10370	9615	1.08	9511	70	12	22	70	64	81	12
BRCA2.67	FEMALE	96.2	97.6	139.452	99.3	98.1	91.1	73.9	47.6	99.5	98.5	92.6	77.8	53.8	2.148	3.101	10409	9484	1.1	9379	73	11	21	76	61	69	17
BRCA2.68	FEMALE	96.2	97.7	141.08	99.3	98.1	91.2	74.5	48.7	99.5	98.5	92.7	78.1	54.3	2.149	3.09	10359	9478	1.09	9371	81	9	17	70	70	90	17
BRCA2.69	FEMALE	96.7	98	153.562	99.4	98.5	93.6	81.5	60.2	99.5	98.7	94.5	83.8	64.6	2.141	3.033	10285	9496	1.08	9395	74	10	17	73	71	63	18
BRCA2.70	FEMALE	96.3	97.7	142.324	99.3	98.2	91.7	75.5	50	99.5	98.5	93	79	55.9	2.146	3.077	10261	9468	1.08	9371	72	9	16	80	56	65	19
BRCA2.71	FEMALE	96.8	98.3	159.798	99.4	98.5	94	83	63.5	99.6	99	95.7	86.9	70.3	2.144	3.064	10377	9526	1.09	9416	78	12	20	76	77	74	15
BRCA2.72	FEMALE	96.7	98.3	161.052	99.4	98.5	94.1	83.5	64.9	99.6	98.8	95	85.6	68.7	2.138	3.052	10357	9595	1.08	9494	67	12	22	82	61	65	19
BRCA2.74	FEMALE	96.8	98.3	161.446	99.4	98.5	94.2	83.9	65.5	99.6	98.9	95.4	86.7	70.6	2.139	3.028	10428	9523	1.1	9423	70	10	20	74	62	65	21
BRCA2.75	FEMALE	96.7	98.3	156.752	99.3	98.4	93.7	82	61.8	99.6	99	95.4	86	68.8	2.141	3.029	10388	9632	1.08	9529	77	9	17	75	64	79	20
BRCA2.76	FEMALE	96.8	98.3	160.815	99.4	98.5	94.2	83.6	64.7	99.6	98.9	95.4	86.5	70	2.143	3.036	10434	9527	1.1	9430	73	8	16	80	70	70	24
BRCA2.77	FEMALE	96.8	98.1	158.218	99.3	98.5	93.9	82.7	62.7	99.6	98.8	94.7	84.4	65.8	2.138	3.033	10457	9570	1.09	9458	81	12	19	67	63	71	23
BRCA2.78	FEMALE	96.8	98.3	159.205	99.4	98.5	93.9	82.7	62.9	99.6	98.9	95	85.1	67.2	2.139	3.099	10500	9586	1.1	9475	80	10	21	64	69	66	20
BRCA2.79	FEMALE	96.8	98.3	160.118	99.4	98.5	94.1	83.4	64.3	99.6	98.8	94.9	85.2	67.7	2.14	3.081	10612	9760	1.09	9656	77	10	17	67	63	71	21
BRCA2.80	FEMALE	96.8	98.4	161.214	99.4	98.5	94.2	83.7	65.2	99.6	99	95.7	87.3	71.6	2.143	3.037	10631	9812	1.08	9704	74	13	21	75	60	68	22
BRCA2.81	FEMALE	96.8	98.2	155.6	99.4	98.5	93.7	82	61.1	99.6	98.9	94.8	84.5	65.7	2.141	3.107	10434	9470	1.1	9364	80	8	18	76	80	81	19
BRCA2.82	FEMALE	96.7	98.2	156.209	99.3	98.4	93.6	81.9	61.3	99.6	98.9	95.1	85.4	67.6	2.14	3.044	10408	9531	1.09	9428	73	12	18	82	69	73	26
BRCA2.84	FEMALE	96.7	98.2	154.85	99.4	98.5	93.7	81.6	60.2	99.6	98.9	95	85	66.2	2.143	3.025	10259	9436	1.09	9322	82	13	19	63	67	70	25
BRCA2.85	FEMALE	96.8	98.4	161.627	99.4	98.6	94.4	84.2	65.7	99.6	99	95.4	86.6	70.3	2.142	3.083	10496	9526	1.1	9415	80	13	18	65	66	60	19
BRCA2.86	FEMALE	96.8	98.3	159.921	99.4	98.5	94.1	83.6	64.9	99.6	98.9	95.3	86.3	69.9	2.139	3.037	10420	9513	1.1	9413	72	8	20	73	65	63	19
BRCA2.87	FEMALE	96.8	98.3	159.517	99.4	98.5	94.2	83.5	64.5	99.6	98.9	95.3	86.3	69.7	2.139	3.053	10376	9710	1.07	9612	69	10	19	70	60	69	25
BRCA2.88	FEMALE	96.8	98.4	158.979	99.4	98.6	94.2	83.3	63.6	99.6	99	95.5	86.5	69.6	2.142	3.02	10263	9472	1.08	9371	70	11	20	73	67	76	21
BRCA2.90	FEMALE	96.8	98.2	157.042	99.4	98.5	94	82.7	62.6	99.6	98.8	94.8	84.9	66.6	2.142	3.083	10326	9396	1.1	9287	75	16	18	73	75	62	24
BRCA2.91	FEMALE	96.9	98.3	159.714	99.4	98.6	94.4	83.6	63.6	99.6	99	95.6	86.6	69.3	2.139	3.009	10543	9633	1.09	9537	64	9	23	67	58	72	24
BRCA2.92	FEMALE	96.8	98.2	160.442	99.4	98.6	94.4	84	65	99.6	98.8	94.8	85.2	67.5	2.138	3.106	10417	9533	1.09	9434	70	12	17	79	64	73	22
BRCA2.93	FEMALE	96.8	98.2	155.989	99.4	98.5	93.8	82.2	61.5	99.6	98.8	94.7	84.3	65.5	2.141	3.012	10307	9502	1.08	9384	80	14	24	73	75	77	28
BRCA2.94	FEMALE	96.4	97.7	164.825	99.3	98.2	92.6	80.1	61	99.5	98.4	93.3	81.7	63.4	2.14	3.101	10903	9956	1.1	9839	84	13	20	88	76	80	25
BRCA2.95	FEMALE	96.7	98.1	164.092	99.3	98.4	93.8	82.7	63.7	99.6	98.7	94.5	84.5	67.1	2.144	3.096	10421	9621	1.08	9523	70	12	16	72	69	83	23
BRCA2.96	FEMALE	96.4	97.9	158.243	99.3	98.1	92	78.2	57.5	99.5	98.6	93.4	81	61.6	2.144	3.073	10432	9640	1.08	9551	63	10	16	64	68	77	24
BRCA2.97	FEMALE	96.4	97.8	162.661	99.3	98.2	92.3	79	58.9	99.5	98.5	93.3	81.2	62.4	2.145	3.059	12839	11343	1.13	11232	72	16	23	83	76	94	17
BRCA2.98	FEMALE	96.5	97.8	162.041	99.3	98.2	92.7	80	60	99.5	98.5	93.3	81.2	61.9	2.142	3.072	13071	11540	1.13	11427	80	11	22	100	70	87	27
BRCA2.99	FEMALE	96.8	98.1	167.027	99.4	98.6	94.4	84.1	65.8	99.6	98.7	94.6	85	68.1	2.143	3.034	10309	9522	1.08	9415	77	10	20	65	67	86	21
BRCA2.100	FEMALE	96.5	97.7	163.626	99.3	98.2	92.6	79.9	60.2	99.5	98.5	93.3	81.4	62.6	2.14	3.073	10421	9350	1.11	9250	72	11	17	77	70	68	22
BRCA2.101	FEMALE	96.4	97.8	159.267	99.3	98	91.7	77.9	57.4	99.5	98.4	92.8	79.9	60.4	2.145	3.036	10244	9455	1.08	9350	79	12	14	72	71	74	25
BRCA2.102	FEMALE	96.5	98	164.178	99.3	98.2	92.4	79.5	60.1	99.5	98.6	93.8	82.4	64.5	2.142	3.041	10500	9390	1.12	9283	77	11	19	62	62	63	14
BRCA2.103	MALE	96.8	98	163.281	99.3	98.1	92	79.1	60.3	99.5	98.6	93.9	83	64.7	2.142	3.035	10270	9379	1.09	9270	71	13	25	75	63	67	18
BRCA2.104	FEMALE	95.9	97.2	146.725	99.2	97.7	89.7	72.3	48.9	99.4	98.1	90.8	74.7	52.3	2.147	3.073	12721	11500	1.11	11393	73	13	21	89	64	88	12
BRCA2.105	FEMALE	96.4	97.9	160.976	99.3	98.1	91.9	78.4	58.3	99.5	98.6	93.5	81.5	62.9	2.146	3.078	10333	9349	1.11	9256	70	9	14	62	57	68	17

Table S1 continued

Sample_ID	CGI_Gender	Percent of Genome Fully Sequenced	Percent of Exome Fully Sequenced	Total Sequence (Gb)	Percent of Genome Coverage					Percent of Exome Coverage					Transition/Transversion Ratio		Synonymous SNVs	Nonsynonymous SNVs	Synonymous/ Nonsynonymous Ratio	Missense SNVs	Nonsense SNVs	Nonstop SNVs	Misstart SNVs	Splice SNVs	Frameshift Insertion	Frameshift Deletion	Frameshift Substitution
					At least 5x	At least 10x	At least 20x	At least 30x	At least 40x	At least 5x	At least 10x	At least 20x	At least 30x	At least 40x	Genome	Exome											
**Median of All Samples**		**96.5**	**97.8**	**161.894**	**99.3**	**98.1**	**92.15**	**78.85**	**59.3**	**99.5**	**98.5**	**93.5**	**82**	**63.55**	**2.143**	**3.0715**	**10391.5**	**9533**	**1.09**	**9433**	**72.5**	**11**	**18**	**72.5**	**66.5**	**72**	**20**
UTSW1	FEMALE	97.4	98.7	301.95	99.5	99	97.2	93.7	88	99.7	99.4	98.3	96	91.9	2.134	3.001	10566	9769	1.08	9652	79	16	22	89	82	72	22
UTSW2	FEMALE	96.4	97.7	160.39	99.3	98.1	91.9	78.2	57.9	99.5	98.3	92.5	79.5	59.9	2.143	3.028	10510	9641	1.09	9538	70	12	21	82	76	80	18
UTSW3	MALE	96.6	97.7	154.902	99.2	97.8	90	74.6	53.8	99.5	98.4	92.7	79.8	59.7	2.143	3.083	10466	9472	1.1	9368	69	11	24	78	66	71	23
UTSW4	MALE	97	98	164.695	99.3	98.3	92.7	80.6	62.5	99.5	98.6	94.2	84.1	66.8	2.139	3.101	10789	9925	1.09	9823	75	10	17	84	68	76	24
UTSW5	FEMALE	96.3	97.7	157.554	99.2	98	91.4	77.1	56.4	99.5	98.4	92.4	79.1	59.2	2.144	3.032	10243	9444	1.08	9347	72	14	11	67	65	76	17
UTSW6	MALE	96.7	98	163.02	99.2	97.9	91.1	77.3	58.2	99.5	98.6	94	83.2	65.4	2.142	3.083	10644	9674	1.1	9591	55	12	16	76	69	86	23
UTSW7	FEMALE	96.6	97.9	168.507	99.3	98.1	92.4	80.1	61.8	99.5	98.5	93.9	83.4	67.4	2.143	3.07	10449	9565	1.09	9466	68	13	18	76	73	66	22
UTSW8	MALE	96.6	97.8	156.15	99.2	97.8	90.2	74.9	54.3	99.5	98.5	93.1	80.5	60.8	2.14	3.152	10584	9803	1.08	9694	85	9	15	85	63	72	17
UTSW9	FEMALE	96.4	97.5	158.223	99.3	98.1	92.2	78.8	57.9	99.4	98.3	92.5	79.4	59	2.143	3.052	10287	9313	1.1	9221	67	10	15	67	72	70	18
UTSW10	FEMALE	96.5	97.9	163.649	99.3	98.2	92.5	79.7	60.1	99.5	98.5	93.3	81.3	62.6	2.141	3.046	10392	9551	1.09	9439	77	15	20	76	71	72	24
UTSW11	FEMALE	96.5	98	161.501	99.3	98.1	92.1	78.7	58.5	99.5	98.6	93.7	81.8	63.3	2.143	3.113	10364	9315	1.11	9218	68	11	18	70	60	64	22
UTSW12	FEMALE	96.4	97.7	155.906	99.2	98	91.7	77.6	56.2	99.5	98.4	92.7	79.5	58.9	2.142	3.026	10255	9514	1.08	9409	75	11	19	63	59	68	21
UTSW13	FEMALE	96.5	97.9	160.164	99.3	98.1	92.2	78.9	58.7	99.5	98.5	93.3	80.9	61.6	2.145	3.103	10891	9828	1.11	9712	88	11	17	82	72	57	27
UTSW14	FEMALE	96.2	97.6	157.478	99.2	98	91.3	76.9	55.9	99.5	98.4	92.8	80	60.5	2.147	3.064	10214	9444	1.08	9341	71	11	21	69	61	57	17
UTSW15	FEMALE	96	97.8	160.906	99.2	97.8	90.9	76.5	56.2	99.5	98.5	93.4	81.7	63.1	2.151	3.106	10299	9584	1.07	9482	73	12	17	65	73	79	20
UTSW16	MALE	96.7	97.7	161.752	99.2	98	91.5	78.3	59.3	99.5	98.4	93.4	82.1	63.7	2.142	3.094	10488	9529	1.1	9413	85	9	22	88	60	74	29
UTSW17	FEMALE	96.5	97.9	162.393	99.3	98.1	92.3	79.2	59.3	99.5	98.5	93.4	81.4	62.5	2.148	3.01	10416	9498	1.1	9406	63	9	20	66	71	67	16
UTSW18	FEMALE	96.5	98	163.081	99.3	98.1	92.2	79.1	59.3	99.5	98.6	93.7	82.1	63.9	2.141	3.085	10294	9530	1.08	9427	75	10	18	67	68	71	18
UTSW19	FEMALE	96.4	97.9	159.446	99.2	98	91.7	77.8	57.3	99.5	98.6	93.6	81.7	63	2.143	3.042	10237	9538	1.07	9444	65	12	17	75	69	76	14
UTSW20	FEMALE	96.5	98.1	162.254	99.3	98.1	92.3	79.5	59.9	99.6	98.7	94.3	83.5	65.9	2.145	3.002	10333	9551	1.08	9449	76	8	18	68	70	74	23
UTSW21	FEMALE	96.3	97.6	154.033	99.2	97.9	90.8	75.5	53.8	99.5	98.3	92.3	78.5	58.1	2.145	3.083	10388	9464	1.1	9343	79	18	24	70	60	69	12
UTSW22	FEMALE	96.6	98	179.191	99.3	98.2	93.2	82.5	66.1	99.5	98.6	94.5	85.3	70.8	2.143	3.125	10396	9543	1.09	9444	71	10	18	71	66	67	19
UTSW23	MALE	96.6	97.9	157.824	99.2	97.8	90.2	75.2	55.1	99.5	98.5	93.3	81.2	62.1	2.144	3.047	10267	9466	1.08	9362	74	12	18	70	66	80	20
UTSW24	FEMALE	96.4	97.7	159.071	99.3	98	91.7	77.9	57.3	99.5	98.3	92.3	78.9	58.8	2.143	3.058	10350	9427	1.1	9338	60	10	19	70	52	70	17
UTSW25	MALE	96.6	97.7	159.167	99.2	97.9	90.6	76.1	56.5	99.5	98.5	93.3	81.4	62.7	2.141	3.079	10803	9748	1.11	9641	73	13	21	79	56	72	18
UTSW26	FEMALE	96.4	97.7	162.341	99.3	98.1	92.2	79.2	59.4	99.5	98.5	93.2	81.2	62.4	2.145	3.031	10602	9633	1.1	9532	72	12	17	72	66	63	19
UTSW27	FEMALE	96.4	97.9	160.75	99.3	98.1	92	78.3	58.1	99.5	98.6	93.8	82.1	64	2.146	3.015	10308	9450	1.09	9357	66	9	18	76	68	72	15
UTSW28	FEMALE	96.5	97.8	159.013	99.3	98.1	92.1	78.6	58.3	99.5	98.4	92.9	80.2	60.7	2.14	3.05	10510	9734	1.08	9633	75	9	17	69	66	74	24
UTSW30	MALE	96.6	97.8	159.214	99.2	97.8	90.2	75.4	55.7	99.5	98.4	93	80.7	62	2.142	3.097	10353	9447	1.1	9345	72	9	21	65	82	74	22
UTSW31	MALE	96.5	97.6	160.569	99.2	97.9	90.6	76.1	56.5	99.5	98.5	93.3	81.5	63.1	2.144	3.164	12844	11370	1.13	11244	92	15	19	94	62	76	22
UTSW32	MALE	96.5	97.8	160.629	99.2	97.8	90.4	76.1	56.8	99.5	98.5	93.6	82.2	64.1	2.145	3.105	10323	9523	1.08	9425	60	16	22	68	59	66	15
UTSW33	MALE	96.6	97.7	160.746	99.2	97.8	90.6	76.3	57	99.5	98.5	93.3	81.6	63.2	2.14	3.043	10491	9656	1.09	9561	67	10	18	67	69	66	23
UTSW34	MALE	96.6	97.7	158.695	99.2	97.8	90.4	75.7	55.6	99.5	98.4	92.9	80.3	61	2.143	3.117	10127	9308	1.09	9201	76	11	20	75	69	67	23
UTSW35	FEMALE	96.4	97.5	157.09	99.3	98.1	92	78	56.7	99.4	98.2	92	78	56.9	2.141	3.142	10700	9829	1.09	9712	88	10	19	84	64	72	29
UTSW36	FEMALE	96.3	97.8	162.88	99.3	98.1	92.2	79.1	59.4	99.5	98.6	93.7	82.2	64.1	2.145	3.116	12693	11496	1.1	11378	81	12	25	99	64	96	19
UTSW37	FEMALE	96.4	97.9	162.036	99.3	98.1	92.2	79	59	99.6	98.7	94.1	82.8	64.6	2.146	3.158	10737	9661	1.11	9563	71	10	17	74	59	64	14
UTSW38	MALE	96.6	97.9	163.394	99.2	97.9	90.9	77.2	58.2	99.5	98.6	93.9	82.9	65.4	2.141	3.117	10580	9737	1.09	9640	70	11	16	74	67	74	20
UTSW39	FEMALE	96.4	97.4	156.244	99.3	98.1	91.7	77.2	55.6	99.4	98.2	91.8	77.6	56.5	2.145	3.11	10284	9416	1.09	9329	65	9	13	67	74	85	22
UTSW40	FEMALE	96.4	97.4	157.333	99.3	98.1	91.9	77.9	56.8	99.4	98.1	91.9	77.9	57.1	2.14	3.067	10337	9422	1.1	9323	71	9	19	75	66	81	13
UTSW41	MALE	96.5	97.6	151.739	99.2	97.6	89.1	72.5	51	99.5	98.4	92.5	78.9	58.6	2.143	3.021	10230	9536	1.07	9434	75	9	18	64	66	66	23
UTSW42	FEMALE	96	97.3	147.802	99.2	97.8	90.1	73.5	50.1	99.4	98.1	91.2	75.7	53.2	2.148	3.11	13022	11469	1.14	11357	77	12	23	96	65	92	15
UTSW43	FEMALE	96.3	97.8	155.121	99.2	98	91.2	76.3	54.7	99.5	98.6	93.1	80.1	60.3	2.145	3.089	10416	9590	1.09	9489	69	11	21	72	66	70	19
UTSW44	FEMALE	96.4	97.6	156.286	99.3	98	91.5	76.9	55.5	99.5	98.4	92.5	79	58.6	2.143	3.113	10340	9296	1.11	9193	74	14	15	69	62	72	16
UTSW45	FEMALE	96.4	97.6	157.974	99.3	98.1	92.1	78.6	57.8	99.4	98.3	92.5	79.3	59.1	2.144	3.099	10277	9341	1.1	9230	72	13	26	68	70	79	12
UTSW46	FEMALE	96.6	98.1	181.499	99.3	98.2	93.1	82.2	65.8	99.5	98.7	94.8	86	72.2	2.142	3.054	10464	9524	1.1	9438	58	9	19	66	82	72	21
UTSW48	FEMALE	96.4	97.7	160.6	99.3	98.1	92	78.5	58.1	99.5	98.5	93.4	81.4	62.6	2.143	3.057	10381	9603	1.08	9505	73	8	17	69	62	78	17
UTSW49	FEMALE	96.4	97.9	161.341	99.3	98.1	92	78.6	58.6	99.5	98.6	93.7	82	63.7	2.145	3.103	10391	9504	1.09	9405	74	7	18	75	66	73	12
UTSW50	FEMALE	96.3	97.8	160.116	99.3	98.1	92	78.3	58	99.5	98.6	93.7	82	63.4	2.146	3.13	11936	10658	1.12	10537	82	15	24	82	64	76	19
UTSW51	FEMALE	96.6	98.1	164.616	99.3	98.1	92.5	80.1	60.9	99.5	98.7	94.1	83	65.3	2.143	3.054	10289	9464	1.09	9376	61	11	16	82	63	64	13
UTSW52	FEMALE	96.6	98.1	168.108	99.3	98.3	93	81.3	62.9	99.6	98.7	94.3	83.9	66.8	2.143	3.045	10376	9457	1.1	9357	72	10	18	77	66	73	18
UTSW53	MALE	96.8	98.1	167.984	99.2	98	91.4	78.3	60.1	99.6	98.7	94.3	83.8	66.9	2.141	2.979	10341	9537	1.08	9432	77	10	18	65	62	74	21
UTSW54	FEMALE	96.6	98	167.415	99.3	98.2	92.8	80.8	62.3	99.5	98.6	94	83.2	65.8	2.14	3.115	10696	9754	1.1	9652	73	7	22	84	62	92	22
UTSW55	FEMALE	96.5	97.8	163.584	99.3	98.2	92.3	79.3	59.5	99.5	98.4	93.1	81.1	62.4	2.142	3.083	10431	9527	1.09	9424	72	11	20	70	66	82	22
UTSW56	FEMALE	96.6	98	181.543	99.3	98.2	93.1	82.1	65.5	99.5	98.7	94.5	85.4	71.1	2.143	3.056	10565	9659	1.09	9565	67	7	20	74	73	78	24
UTSW57	FEMALE	96.4	97.9	172.352	99.3	98.1	92.3	80.1	62	99.5	98.6	94	84	68.6	2.145	3.068	10231	9522	1.07	9418	75	12	17	72	73	79	24
UTSW58	FEMALE	96.4	97.9	156.913	99.3	98	91.7	77.5	56.4	99.5	98.5	93.1	80.4	60.5	2.146	3.08	10166	9413	1.08	9326	62	8	17	66	72	70	22
UTSW60	MALE	96.7	98	174.488	99.3	98	91.5	78.7	61.3	99.5	98.7	94.5	85.1	70.3	2.141	3.077	10381	9484	1.09	9393	67	10	14	82	64	79	18
UTSW61	FEMALE	96.5	98	172.83	99.3	98.2	92.4	80.3	62.5	99.5	98.7	94.5	84.9	70	2.144	3.034	10394	9397	1.11	9296	74	9	18	79	69	54	22
UTSW62	FEMALE	96.5	97.9	175.636	99.3	98.1	92.7	81.3	64.3	99.5	98.7	94.5	85.1	70.4	2.144	3.049	10272	9465	1.09	9365	67	13	20	76	50	71	15
UTSW63	FEMALE	96.5	97.9	174.518	99.3	98.1	92.5	81	64	99.5	98.6	94.2	84.6	69.8	2.144	3.038	10381	9572	1.08	9469	73	9	21	72	66	66	21
UTSW64	FEMALE	96.4	97.7	156.475	99.2	98	91.4	77.1	55.9	99.5	98.3	92.5	79.1	59	2.143	3.084	10373	9520	1.09	9412	77	10	21	74	68	83	20
UTSW65	FEMALE	96.4	97.9	174.149	99.3	98.1	92.3	80.4	62.8	99.5	98.6	94	84.1	69	2.145	3.071	10405	9630	1.08	9514	83	11	22	67	72	64	15
UTSW66	FEMALE	96.3	97.9	174.055	99.2	98	91.8	79.3	61.7	99.5	98.6	94	84.1	69.4	2.147	3.057	10744	9707	1.11	9595	85	13	14	66	68	70	18
UTSW67	FEMALE	96.5	98	179.903	99.2	98.1	92.6	81.3	64.5	99.5	98.7	94.5	85.4	71.2	2.142	3.107	10029	9405	1.07	9309	72	9	15	64	67	67	19
UTSW68	FEMALE	96.8	97.4	156.861	99.4	98.5	93.8	82.5	62.7	99.4	98.2	92.4	80	59.5	2.135	3.072	10141	9506	1.07	9408	71	9	18	78	62	71	20
UTSW69	FEMALE	96.8	97.5	155.352	99.4	98.5	93.6	82	61.7	99.4	98.3	92.5	79.9	59.1	2.135	3.03	10449	9644	1.08	9528	85	10	21	70	66	79	20
UTSW70	FEMALE	96.9	97.9	165.555	99.4	98.6	94.5	84.7	67.1	99.5	98.5	93.9	83.6	65.8	2.137	3.07	10243	9461	1.08	9355	76	13	17	66	72	70	21
UTSW71	FEMALE	97	97.8	169.102	99.4	98.6	94.8	85.7	69.3	99.5	98.4	93.5	83.1	65.6	2.137	3.036	10191	9496	1.07	9383	79	12	22	73	71	73	15
UTSW72	FEMALE	96.6	98	180.752	99.3	98.2	93.1	82	65.5	99.5	98.7	94.8	86.1	72.3	2.142	3.053	10446	9653	1.08	9544	75	15	19	83	73	73	15
UTSW73	FEMALE	97	97.7	170.646	99.4	98.7	95.1	86.6	71	99.5	98.4	93.6	83.5	66.7	2.134	3.097	10307	9463	1.09	9361	68	14	20	70	59	56	23
UTSW74	MALE	96.8	98.2	178.913	99.3	98	91.9	79.7	63.2	99.6	98.9	95.4	87.1	73.8	2.146	3.044	10355	9538	1.09	9448	64	9	17	74	72	73	22
UTSW75	FEMALE	96.5	97.9	177.852	99.2	98.1	92.6	81.2	64.5	99.5	98.6	94.3	84.9	70.5	2.145	3.071	10481	9612	1.09	9512	71	10	19	61	67	83	18
UTSW76	FEMALE	97	97.8	170.754	99.4	98.7	94.9	86	70	99.5	98.4	93.4	83.1	65.8	2.135	3.078	10464	9630	1.09	9537	68	11	14	76	78	73	17
UTSW77	FEMALE	96.9	97.7	169.276	99.4	98.6	94.7	85.4	69	99.5	98.4	93.3	82.8	65.3	2.137	3.112	10438	9524	1.1	9417	74	14	19	70	66	70	26
UTSW78	FEMALE	96.6	97.7	161.341	99.3	98.2	92.2	78.9	59.3	99.5	98.4	92.9	80.6	62.3	2.145	3.096	10522	9540	1.1	9438	70	12	20	83	74	85	24
UTSW79	FEMALE	97	97.8	169.011	99.4	98.7	94.9	85.8	69.3	99.5	98.5	93.7	83.6	66.4	2.138	3.051	10398	9457	1.1	9367	60	13	17	60	77	62	21
UTSW80	FEMALE	96.9	97.8	168.565	99.4	98.6	94.8	85.6	68.9	99.5	98.5	94	84.2	67.2	2.141	3.11	13040	11736	1.11	11614	89	15	18	100	72	87	22
UTSW81	MALE	96.6	97.9	157.872	99.2	97.8	90.1	75.1	55	99.5	98.6	93.5	81.5	62.8	2.143	3.071	10095	9527	1.06	9422	72	13	20	73	66	69	19
UTSW82	FEMALE	96.9	97.9	169.769	99.4	98.6	94.8	85.7	69.3	99.5	98.6	94.3	84.7	68.3	2.137	3.111	10405	9510	1.09	9412	72	9	17	71	81	62	15
UTSW83	FEMALE	97	97.9	171.019	99.4	98.7	95.1	86.5	70.7	99.5	98.5	94	84.4	68	2.136	3.055	10310	9581	1.08	9474	78	11	18	77	76	67	32
UTSW84	FEMALE	96.5	97.5	159.2	99.3	98.2	92.2	78.7	58.5	99.5	98.4	92.8	80.5	61.8	2.141	3.108	10412	9548	1.09	9440	79	11	18	74	61	93	17
UTSW85	FEMALE	97	97.8	169.47	99.4	98.7	95	86.2	70.2	99.5	98.5	93.8	84	67.2	2.137	3.066	10268	9463	1.09	9349	78	16	20	71	72	69	23

Table S2. *BRCA1* -population cancer history (n=88)

Patient	BRCA1-variant*	Cancer History**	Gender	Patient	BRCA1-variant*	Cancer History**	Gender
BRCA1.1	2800delAA	Breast-39	F	BRCA1.50	2800delAA	Breast-40	F
BRCA1.2	300T>G	Unaffected-53	F	BRCA1.52	1135insA	Ovarian-58	F
BRCA1.3	5536delC	Breast-54	F	BRCA1.53	5382insC	Breast-60; Esophagus-62	F
BRCA1.4	3937C>T	Unaffected-32	F	BRCA1.55	4987C>G	Breast-47	F
BRCA1.5	4962del15	Unaffected-24	F	BRCA1.56	IVS13+1G>A	Breast-41	F
BRCA1.6	5187A>T	Breast-27	F	BRCA1.57	5382insC	Unaffected-61	M
BRCA1.7	IVS20+1G>A	Unaffected-61	M	BRCA1.58	300T>G	Breast-35	F
BRCA1.8	del exons 1-2	Breast-31	F	BRCA1.59	1294del40	Breast-40; Thyroid-36; Skin-34	F
BRCA1.9	2925del4	Breast-37	F	BRCA1.60	5382insC	Breast-47,49	F
BRCA1.10	3889delAG	Breast-68	F	BRCA1.61	IVS12+22del21insA	Unaffected-24	F
BRCA1.11	2798del4	Unaffected-44	M	BRCA1.62	exon 13 ins 6KB	Breast-38	F
BRCA1.12	3987A>T	Breast-38	F	BRCA1.63	2798del4	Breast-46; Peritoneum-50	F
BRCA1.13	2576delC	Unaffected-43	F	BRCA1.64	IV5S5-11T>G	Breast-34,59	F
BRCA1.15	del exons 9-12	Unaffected-19	F	BRCA1.65	300T>G	Bladder-70	M
BRCA1.16	3600del11	Breast-38	F	BRCA1.66	5410T>C	Breast-32	F
BRCA1.17	2530delAG	Unaffected-54	F	BRCA1.67	187delAG	Breast-36	F
BRCA1.18	3748G>T	Breast-37	F	BRCA1.68	5326T>C	Unaffected-55	F
BRCA1.19	1240delC	Unaffected-66	F	BRCA1.69	2257C>G	Unaffected-43	F
BRCA1.22	2634delC	Unaffected-51	F	BRCA1.71	exon 13 ins 6KB	Breast-32,42	F
BRCA1.23	262delT	Ovarian-63	F	BRCA1.72	del exons 21-24	Breast-43,62; Endometrium-60	F
BRCA1.24	3171ins5	Unaffected-42	F	BRCA1.73	1294del40	Breast-32	F
BRCA1.26	2576delC	Breast-31	F	BRCA1.74	1599C>T	Breast-42,53,55	F
BRCA1.27	3875del4	Unaffected-32	F	BRCA1.75	2576delC	Breast-47	F
BRCA1.28	300T>G	Breast-32	F	BRCA1.77	2722C>G	Breast-74	F
BRCA1.29	1135insA	Breast-38	F	BRCA1.78	exon 13 ins 6KB	Breast-38	F
BRCA1.30	1623del5	Breast-39	F	BRCA1.80	2552delC	Breast-36	F
BRCA1.31	exon 13 ins 6KB	Unaffected-42	F	BRCA1.81	2722C>G	Breast-45,51,65; Skin-65	F
BRCA1.32	187delAG	Breast-48; Peritoneum-59,62	F	BRCA1.83	1081G>A	Breast-32; Fallopian Tube-33	F
BRCA1.33	IVS16+3G>C	Unaffected-52	F	BRCA1.84	3124delA	Unaffected-53	F
BRCA1.34	1294del40	Breast-35	F	BRCA1.85	2722C>G	Unaffected-26	F
BRCA1.36	exon 5-7 deletion	Ovarian-45	F	BRCA1.86	300T>G	Breast-30,32,34	F
BRCA1.40	1294del40	Breast-47,51	F	BRCA1.87	3203del11	Breast-56	F
BRCA1.41	300T>G	Breast-29,48	F	BRCA1.90	2295delC	Breast-28,40,40	F
BRCA1.42	5382insC	Breast-49	F	BRCA1.91	3438G>T	Ovarian-41	F
BRCA1.44	3600del11	Breast-37	F	BRCA1.93	795delT	Unaffected-67	F
BRCA1.46	1547del10	Breast-33	F	BRCA1.94	IVS16+4A>C	Breast-32	F
BRCA1.47	exon 20 deletion	Breast-29,31	F	BRCA1.96	2722C>G	Breast-49	F
BRCA1.48	187delAG	Breast-39; Skin-61,66	F	BRCA1.97	5215G>A	Breast-38	F
BRCA1.49	IVS4-1G>T	Breast-48,49	F	BRCA1.98	187delAG	Unaffected-25	F

*The variant description is as clinically reported (Myriad Genetics, Inc.)

** Age at diagnosis or current age of unaffected

Table S2 continued

Patient	BRCA1-variant*	Cancer History**	Gender	Patient	BRCA1-variant*	Cancer History**	Gender
BRCA1.99	5228T>G	Breast-39	F	BRCA1.106	exon 13 ins 6KB	Breast-51,51; Ovarian-52	F
BRCA1.100	5382insC	Breast-57	F	BRCA1.107	1294del40	Breast-44	F
BRCA1.102	IVS18-2delA	Breast-40	F	BRCA1.108	5382insC	Melanoma-38	F
BRCA1.104	3875del4	Breast-49	F	BRCA1.111	1172+1G>A	Breast-29	F
BRCA1.105	2529C>T	Unaffected-40	F	BRCA1.112	4982C>A	Breast-33	F

*The variant description is as clinically reported (Myriad Genetics, Inc.)

** Age at diagnosis or current age of unaffected

Table S3. *BRCA2* - population cancer history (n=88)

Patient	BRCA2-variant*	Cancer History**	Gender	Patient	BRCA2-variant*	Cancer History**	Gender
BRCA2.1	1638G>T	Unaffected-55	M	BRCA2.48	6503delTT	Breast-46	F
BRCA2.2	7990del3ins2	Unaffected-54	F	BRCA2.49	6174delT	Breast-46	F
BRCA2.3	8395G>C	Unaffected-45	F	BRCA2.50	6672delT	Breast-43	F
BRCA2.4	8878C>T	Breast-39	F	BRCA2.51	6674del5	Breast-35	F
BRCA2.5	3036del4	Breast-43,43	F	BRCA2.52	8096A>G	Breast-32	F
BRCA2.6	999del5	Breast-32,41	F	BRCA2.53	7174del4	Breast-40	F
BRCA2.7	8525delC	Breast-49	F	BRCA2.54	IVS15-1G>A	Ovarian-50	F
BRCA2.9	6174delT	Unaffected-76	F	BRCA2.55	2041insA	Breast-45; Skin-53	F
BRCA2.11	7297delCT	Ovarian-22,22; Thyroid-14	F	BRCA2.56	5270delTG	Breast-46	F
BRCA2.12	5263delA	Breast-30	F	BRCA2.58	5920delG	Breast-44,44	F
BRCA2.13	4101delA	Breast-28,39,55; Skin-49	F	BRCA2.59	6092C>A	Breast-32	F
BRCA2.14	2041insA	Breast-39,39	F	BRCA2.61	8765delAG	Breast-58; Ovarian-55; Colon-52	F
BRCA2.15	3427delA	Testis-25; Ampulla of vater-51	M	BRCA2.63	4512insT	Breast-54; Melanoma-49	F
BRCA2.16	5466insT	Unaffected-43	F	BRCA2.65	1983del5	Melanoma-50	F
BRCA2.17	5578delAA	Breast-41	F	BRCA2.67	9132delC	Breast-34; Melanoma-29	F
BRCA2.18	6620del5	Ovarian-60	F	BRCA2.68	5930delAG	Unaffected-37	F
BRCA2.19	690delAA	Breast-35,45	F	BRCA2.69	3014insT	Unaffected-45	F
BRCA2.21	6137C>A	Breast-55,63	F	BRCA2.70	9382C>T	Unaffected-53	F
BRCA2.22	983del4	Breast-37	F	BRCA2.71	1638G>T	Unaffected-44	F
BRCA2.23	9610C>T	Unaffected-38	F	BRCA2.72	8395G>C	Ovarian-61	F
BRCA2.24	4965C>G	Breast-39	F	BRCA2.74	IVS15-1G>A	Ovarian-62,62	F
BRCA2.25	9345G>A	Breast-42,46	F	BRCA2.75	IVS15-1G>A	Breast-48	F
BRCA2.26	9631delC	Unaffected-28	F	BRCA2.76	5910C>G	Breast-48,58	F
BRCA2.27	9513C>G	Breast-44	F	BRCA2.77	6174delT	Unaffected-24	F
BRCA2.28	8765delAG	Breast-50	F	BRCA2.78	5910C>G	Breast-31	F
BRCA2.29	2060C>A	Breast-43; Ovarian-45	F	BRCA2.79	6174delT	Breast-39; Skin-55	F
BRCA2.31	7990del3ins2	Breast-58; Lung-61	F	BRCA2.80	3036del4	Unaffected-35	F
BRCA2.34	7990del3ins2	Melanoma-48	F	BRCA2.81	7297delCT	Breast-48; Ovarian-52	F
BRCA2.35	6174delT	Skin-64	F	BRCA2.82	5578delAA	Breast-43	F
BRCA2.36	2157delG	Breast-39	F	BRCA2.84	6633del5	Unaffected-41	F
BRCA2.37	6503delTT	Breast-54	F	BRCA2.85	IVS5+1G>T	Unaffected-37	F
BRCA2.38	9631delC	Breast-37	F	BRCA2.86	8237C>T	Breast-73; Peritoneum-74	F
BRCA2.39	9481insA	Unaffected-47	F	BRCA2.87	6503delTT	Unaffected-42	F
BRCA2.40	373G>T	Breast-35	F	BRCA2.88	5301insA	Breast-36	F
BRCA2.41	2028T>G	Breast-50	F	BRCA2.90	4075delGT	Ovarian-49,49	F
BRCA2.42	7297delCT	Unaffected-59	M	BRCA2.91	7297delCT	Unaffected-55	F
BRCA2.43	8096A>G	Unaffected-41	F	BRCA2.92	2157delG	Unaffected-28	F
BRCA2.44	1983del5	Breast-58	F	BRCA2.93	5910C>G	Breast-37,61; Ovarian-56	F

*The variant description is as clinically reported (Myriad Genetics, Inc.)

** Age at diagnosis or current age of unaffected

Table S3 continued

Patient	BRCA2-variant*	Cancer History**	Gender	Patient	BRCA2-variant*	Cancer History**	Gender
BRCA2.94	4150G>T	Breast-56	F	BRCA2.100	6306delAA	Breast-62	F
BRCA2.95	6503delTT	Unaffected-69	F	BRCA2.101	1054del5	Unaffected-40	F
BRCA2.96	8141del5	Unaffected-39	F	BRCA2.102	9371A>T	Breast-36	F
BRCA2.97	3036del4	Breast-45	F	BRCA2.103	5164del4	Breast-58; Testicular-31	M
BRCA2.98	4355del4	Breast-52	F	BRCA2.104	8764A>G	Breast-33	F
BRCA2.99	6503delTT	Ovarian-43	F	BRCA2.105	5645C>A	Unaffected-47	F

*The variant description is as clinically reported (Myriad Genetics, Inc.)

** Age at diagnosis or current age of unaffected

Table S4. Non-*BRCA* population cancer history (n=82)

Patient	Cancer History*	Clinical Testing	Gender	Patient's Family History of Cancer
UTSW1	Breast-38; Thyroid-38	BRCA, CancerNext and PTEN Negative	F	Colon, Leukemia, Lung
UTSW2	Unaffected-61	BRCA Negative	F	Breast, Colon, Ovarian
UTSW3	Adrenocortical-1	TP53 p.R196X	M	Breast, Lung, Prostate
UTSW4	Papillary multifocal RCC-33	MET and VHL Negative	M	Breast
UTSW5	Breast-48	BRCA Negative	F	Breast, Colon, Liver, Lung
UTSW6	Clear cell RCC-40	VHL Negative	M	none
UTSW7	Breast-35	BreastNext Negative	F	Breast, Colon, Lung, Prostate
UTSW8	Colon polyposis-30	MYH and APC Negative	M	Brain, Colon
UTSW9	B-cell lymphoma (DLCBL)-41	Not tested	F	Breast, Lung, Multiple Myeloma, RCC
UTSW10	Unaffected-57	BRCA Not Tested	F	Brain, Breast, Lung, Stomach
UTSW11	Pheo, PGL-53	SDHD Negative	F	Breast, Colon
UTSW12	Colon polyposis-51	APC c.509del4	F	none
UTSW13	Leiomyoma-33	FH c.424_425insTG	F	Breast, Colon, Leiomyoma, RCC
UTSW14	Clear cell RCC, Hb-53	VHL Negative	F	Liver, Lung
UTSW15	Breast-37,50	BRCA Negative	F	Breast, Ovarian
UTSW16	Colon polyposis-53	APC c.5757delT	M	none
UTSW17	Colon-35	Lynch Negative, Syndrome X	F	Colon
UTSW18	Hyperparathyroidism-29	MEN1 Negative	F	Breast, Lymphoma, Pancreatic
UTSW19	Breast-62	PTEN exon 6 dup	F	Brain, Breast, Esophageal, Lymphoma, Ovarian, Stomach
UTSW20	Breast-55	BRCA Negative	F	Breast, Prostate
UTSW21	Unaffected-44	Lynch MSH2 del exons 1-6	F	Colon, Endometrial, Pancreatic, Papillary RCC, Ovarian
UTSW22	Breast-48,56; Pancreatic-58	BRCA and CancerNext Negative	F	Breast, Colon, Ovarian, Prostate, Stomach
UTSW23	Hb-52; Medullary thyroid-53	MEN1, MEN2, VHL and RET Negative	M	Glioblastoma, Melanoma, Ovarian
UTSW24	Melanoma-33; Breast-45	BRCA Negative	F	Colon, Lymphoma
UTSW25	Unaffected-48	Lynch PMS2 p.M622V	M	Brain, Breast
UTSW26	Unaffected-46	Lynch PMS2 p.R802X	F	Brain, Lymphoma
UTSW27	Unaffected-73	Lynch MSH2 del exons 1-6	F	Endometrial, Stomach
UTSW28	Colon-41	MYH frameshift	F	none
UTSW30	Colon polyposis-56	PTEN full deletion	M	Brain, Breast, Colon, Prostate
UTSW31	Colon-52	Lynch MSH6 c.1705_1706delTT	M	Endometrial
UTSW32	Colon-40	Lynch MSH6 p.R911X	M	none
UTSW33	Colon-46	Lynch Negative	M	none
UTSW34	Colon-53	Lynch Negative, Syndrome X	M	Colon
UTSW35	Breast-36	BRCA Negative	F	Breast, Prostate
UTSW36	Vulvar-64; Breast-68	BRCA Negative	F	Breast, Pancreatic, Prostate, Stomach
UTSW37	Breast-27	BRCA and TP53 Negative	F	Endometrial, Ovarian
UTSW38	Papillary RCC-48	FH c.424_425insTG	M	Breast, Leiomyoma
UTSW39	Breast-48	BRCA Negative	F	Breast, Colon, Lung, Ovarian
UTSW40	Unaffected-64	BRCA Negative	F	Bladder, Breast, Lung, Melanoma, Pancreatic, Testicular
UTSW41	Colon-57	Lynch uninformative MLH1 p.R127K	M	Brain, Leukemia, Lung, Lymphoma
UTSW42	Breast-41	BRCA Negative	F	Breast, Colon, Lung, Stomach

* Age at diagnosis or current age of unaffected

Abbreviations: Hb, Hemangioblastoma; PGL, Paraganglioma; Pheo, Pheochromocytoma; RCC, Renal Cell Carcinoma

MyRisk (Myriad); CancerNext, BreastNext, OvaNext, RenalNext (Ambry) are all panels of cancer genes.

Table S4 continued

Patient	Cancer History*	Clinical Testing	Gender	Patient's Family History of Cancer
UTSW43	Breast-42	BRCA Negative	F	Breast
UTSW44	Unaffected-27	Lynch MSH2 p.389X	F	Colon
UTSW45	Colon-26	Lynch MLH1 p.V506A	F	Bladder, Breast, Colon, Endometrial, Leukemia
UTSW46	Colon-50	BRCA and CancerNext Negative	F	Breast, Ovarian, Clear cell RCC
UTSW48	Breast-56	BRCA Negative	F	Breast, Esophageal, Ovarian
UTSW49	Pheo-22; Thyroid-22	RET c.C634R	F	Thyroid
UTSW50	Unaffected-30	BRCA Negative	F	Breast
UTSW51	Unaffected-40	Lynch PMS2 del exon 10	F	Brain, Lung
UTSW52	Unaffected-61	Lynch PMS2 del exon 10	F	Brain, Lung
UTSW53	Unaffected-30	Lynch MSH6 c.3037_3041delAAGAA	M	Colon
UTSW54	Breast-48	BRCA Negative	F	Breast, Endometrial, Stomach
UTSW55	Colon polyposis-26	APC c.3927_3931delAAAGA	F	Colon
UTSW56	Unaffected-55	BRCA and PTEN Negative	F	Breast, Thyroid
UTSW57	Breast-50,54	BRCA Negative	F	Ovarian
UTSW58	Unaffected-59	EGFR p.T790M	F	Lung
UTSW60	Papillary RCC-36; Thyroid-36	RenalNext Negative	M	none
UTSW61	Ovarian-58	BRCA and OvaNext Negative	F	Bladder, Colon, Endometrial, Larynx, Melanoma, Stomach
UTSW62	Breast-70	BRCA and BreastNext Negative	F	Abdominal, Brain, Breast, Endometrial, Liver, Intestinal, Ovarian
UTSW63	Breast-40; Colon-50	BRCA Negative	F	Bladder, Lung, Meningioma
UTSW64	Brain-49	BRCA and TP53 Negative	F	Breast, Colon, Leukemia, Lung, Ovarian, Prostate
UTSW65	Breast-41,51	BRCA Negative	F	Breast, Esophageal, Lung, Pancreatic
UTSW66	Breast-42; Liver-55	BRCA and BreastNext Negative	F	Breast, Lung, Ovarian, Prostate
UTSW67	Breast-49	BRCA, TP53 and BreastNext Negative	F	Breast
UTSW68	Breast-51	BRCA Negative	F	Breast, Colon, Esophageal, Intestinal, Pancreatic
UTSW69	Breast-41	BRCA Negative; BreastNext positive for MUTYH p.G396D	F	Breast, Lymphoma
UTSW70	Breast-47	BRCA Negative	F	Brain, Breast, Liver, Lung, Ovarian
UTSW71	Breast-45,45	BRCA Negative	F	Breast, Melanoma
UTSW72	Breast-65	BRCA Negative	F	Breast, Colon, Endometrial, Esophageal, Melanoma
UTSW73	Renal-36	VHL p.P2L (VUS)	F	Lymphoma, Myeloma
UTSW74	Colon-55; Prostate-55	BRCA Negative	M	Brain, Breast, Colon, Prostate, Renal
UTSW75	Pheo-46	VHL Negative	F	Ovarian, Renal
UTSW76	Breast-47	CHEK2 p.Y424H (VUS)	F	Breast, Colon, Endometrial, Prostate, Thyroid
UTSW77	Breast-66	BRCA Negative	F	Breast, Chordoma, Colon, Ovarian
UTSW78	Breast-54,56	ATM c.2502dupA, RAD50 c.326_329delCAGA	F	Brain, Breast, Colon, Lung, Prostate
UTSW79	Breast-12	TP53 del exon 1	F	Breast, Colon, Osteosarcoma, Rhabdomyosarcoma, Sarcoma
UTSW80	Ovarian-46	BRCA Negative	F	Breast, Lung, Stomach
UTSW81	Glioblastoma-50	TP53 Negative	M	Glioblastoma, Renal
UTSW82	Breast-61; Renal oncocytoma-66	BRCA Negative	F	Breast, Colon, Prostate
UTSW83	Thyroid-31; Ovarian-37	MyRisk Negative	F	Bladder, Breast, Leukemia, Lymphoma, Ovarian
UTSW84	Breast-64; Thyroid-65	BRCA Negative	F	Breast, Prostate
UTSW85	Ovarian-20	MyRisk Negative	F	Breast, Colon, Prostate

* Age at diagnosis or current age of unaffected

Abbreviations: Hb, Hemangioblastoma; PGL, Paraganglioma; Pheo, Pheochromocytoma; RCC, Renal Cell Carcinoma

MyRisk (Myriad); CancerNext, BreastNext, OvaNext, RenalNext (Ambry) are all panels of cancer genes

Table S5. WGS Concordance for *BRCA1* and *BRCA2* Diagnostic Variants

	CNVs*	IVS**	SNVs	Indels	SNVs+Indel
**BRCA1**	10	3	27/29	40/46	67/75
			93.10%	86.96%	89.33%
**BRCA2**	0	0	29/29	49/59	78/88
			100%	83.05%	88.64%
**All BRCA**	10	3	56/58	89/105	145/163***
			96.55%	84.76%	88.96%

* Copy number variants (CNV) were not included in the analysis

** Variants in the intronic intervening sequence (IVS) not affecting the canonical splice site were not included in the analysis

*** All discordant variants, excluding one in-frame deletion identified by WGS, were detected by WGS but considered low quality

Table S6. *BRCA1* mutations identified by both clinical testing and WGS

Mutation Type	Protein Change	# Cases	WGS*
Small Insertion/Deletion			
Frameshift			
795delT	p.C179fs	1	-
1135insA	p.V293fs	2	+
1240delC	p.T327fs	1	+
1294del40	p.L345fs	5	+
1547del10	p.H429fs	1	+
1623del5	p.L455fs	1	+
187delAG	p.E23fs	4	+/-
2295delC	p.L679fs	1	+
2530delAG	p.Q757fs	1	+
2552delC	p.K765fs	1	-
2576delC	p.D774fs	3	+
262delT	p.M1fs	1	-
2634delC	p.H792fs	1	+
2798del4	p.K846fs	2	+
2800delAA	p. K847fs	2	+
2925del4	p.D889fs	1	+
3124delA	p.N1002fs	1	+
3171ins5	p.N1018fs	1	+
3203del11	p.N1029fs	1	+
3600del11	p.E1114fs	2	+
3875del4	p.S1206fs	2	+
3889delAG	p.E1210fs	1	+
5382insC	p.Q1709fs	6	+
5536delC	p.P1759fs	1	-
In-frame			
4962del15	p.1556_1560delPQLKV	1	+
Point Mutation			
Missense (amino acid change)			
300T>G	p.C61G	6	+
4987C>G	p.A1576G	1	+
4982C>A	p.A1708E	1	+
5215G>A	p.R1652Q	1	+
5326T>C	p.V1689A	1	+
5410T>C	p.L1717P	1	+
Point Mutation (cont.)			
Nonsense (stop gain)			
1081G>A	p.W274*	1	+
1599C>T	p.Q447*	1	+
2257C>G	p.S713*	1	+
2529C>T	p.Q757*	1	+
2722C>G	p.S821*	4	+
3438G>T	p.E1060*	1	+
3748G>T	p.E1250*	1	+
3937C>T	p.Q1313*	1	+
3987A>T	p.K1290*	1	+
5187A>T	p.K1690*	1	-
5228T>G	p.Y1656*	1	+
Intronic			
Canonical Splice Site			
1172+1G>A	--	1	+
IVS4-1G>T	--	1	-
IVS12+22del21insA	--	1	+
IVS13+1G>A	--	1	+
IVS18-2delA	--	1	+
IVS20+1G>A	--	1	+
Intervening Sequence			
IVS5-11T>G	--	1	N/A
IVS16+3G>C	--	1	N/A
IVS16+4A>C	--	1	N/A
Large Insertion/Deletion			
exon 5-7 del	--	1	N/A
exon 13 ins 6kb	--	5	N/A
exon 20 del	--	1	N/A
del exons 1-2	--	1	N/A
del exons 9-12	--	1	N/A
del exons 21-24	--	1	N/A

* Whole Genome Sequence (WGS); intervening sequence variants and copy number variants were not investigated (N/A); mutations identified (+) and not identified (-) by WGS are shown

Table S7. *BRCA2* mutations identified by both clinical testing and WGS

Mutation Type	Protein Change	# Cases	WGS*	Mutation Type	Protein Change	# Cases	WGS*
Small Insertion/Deletion				***Small Insertion/Deletion (cont.)***			
Frameshift				*Frameshift (cont.)*			
690delAA	p.D156fs	1	+	8141del5	p.F2638fs	1	+
983del4	p.D252fs	1	+	8525delC	p.T2766fs	1	+
999del5	p.N257fs	1	+	8765delAG	p.E2846fs	2	+
1054del5	p.V276fs	1	+	9132delC	p.V2969fs	1	-
1983del5	p.K585fs	2	+	9481insA	p.T3085fs	1	-
2041insA	p.I605fs	2	-	9631delC	p.L3135fs	2	+
2157delG	p.R645fs	2	+	***Point Mutation***			
3014insT	p.L929fs	1	-	*Missense*			
3036del4	p.A938fs	3	+	8096A>G	p.H2623R	2	+
3427delA	p.T1067fs	1	+	8237C>T	p.S2670L	1	+
4075delGT	p.V1283fs	1	+	8395G>C	p.D2723H	2	+
4101delA	p.Q1291fs	1	+	8764A>G	p.S2922G	1	+
4355del4	p.G1376fs	1	+	9345G>A	p.P3039P	1	+
4512insT	p.Q1429fs	1	-	9371A>T	p.N3124I	1	+
5164del4	p.E1646fs	1	+	9382C>T	p.R3052W	1	+
5263delA	p.T1679fs	1	-	9513C>G	p.D3095E	1	+
5270delTG	p.V1681fs	1	+	*Nonsense*			
5301insA	p.W1692fs	1	-	373G>T	p.E49*	1	+
5466insT	p.N1747fs	1	+	1638G>T	p.E471*	2	+
5578delAA	p.N1784fs	2	+	2028T>G	p.Y600*	1	+
5920delG	p.D12898fs	1	+	2060C>A	p.S611*	1	+
5930delAG	p.E1901fs	1	+	4150G>T	p.E1308*	1	+
6174delT	p.S1982fs	5	+	4965C>G	p.Y1655*	1	+
6306delAA	p.E2028fs	1	+	5645C>A	p.S1882*	1	+
6503delTT	p.L2092fs	5	+	5910C>G	p.Y1894*	3	+
6620del5	p.K2131fs	1	+	6092C>A	p.S1955*	1	+
6633del5	p.N2135fs	1	+	6137C>A	p.S1970*	1	+
6672delT	p.I2149fs	1	+	8878C>T	p.Q2960*	1	+
6674del5	p.I2149fs	1	+	9610C>T	p.R3128*	1	+
7174del4	p.I2315fs	1	-	***Intronic***			
7297delCT	p.L2357fs	4	+/-	IVS5+1G>T	--	1	+
7990del3ins2	p.I2588fs	3	+	IVS15-1G>A	--	3	+

* Whole Genome Sequence (WGS); mutations identified (+) and not identified (-) by WGS are shown

Table S8. Distribution of nonsynonymous potentially pathogenic variants

	In-frame Deletion	In-frame Insertion	Missense	Splice Disrupt	Frameshift	Nonsense	Misstart	Nonstop	All
Number of Variants*	12	3	577	11	45	46	1	0	695
Percent Novel**	100%	100%	36.40%	100%	100%	89.13%	0%	0%	46.33%

* *BRCA1* and *BRCA2* pathogenic variants were not included

** Not present in the ESP6500 database

Table S9. Number of potentially pathogenic variants and individuals in cancer-associated genes in the *BRCA1/2-* cohort

Gene	All Variants		LoF Variants		Gene	All Variants		LoF Variants		Gene	All Variants		LoF Variants	
	Cases	Variants	Cases	Variants		Cases	Variants	Cases	Variants		Cases	Variants	Cases	Variants
ATM	22	18	2	2	BAP1	3	3	0	0	XRCC2	1	1	0	0
BRCA2*	31	17	0	0	NF1	4	3	0	0	XRCC3	1	1	0	0
APC	16	13	0	0	FLCN	3	3	0	0	FH	1	1	0	0
BRCA1*	22	11	0	0	NTRK1	2	3	0	0	BLM	0	0	0	0
TSC2	12	10	0	0	MRE11A	4	2	0	0	CDH1	0	0	0	0
TP53BP1	13	10	0	0	GALNT12	2	2	0	0	EGFR	0	0	0	0
CREBBP	9	8	0	0	EPCAM	2	2	0	0	CDKN1A	0	0	0	0
ATR	12	7	0	0	GREM1	3	2	0	0	KIT	0	0	0	0
MLH3	8	7	0	0	MUTYH	2	2	0	0	VHL	0	0	0	0
SMO	8	6	0	0	GEN1	37	2	36	1	WT1	0	0	0	0
PMS2	13	6	0	0	NBN	2	2	0	0	BRIP1	0	0	0	0
RB1	7	6	0	0	RAD51	2	2	0	0	CDC73	0	0	0	0
MSH6	9	6	0	0	SDHB	2	2	0	0	CHEK1	0	0	0	0
MSH2	12	5	0	0	ELAC2	2	2	0	0	HOXB13	0	0	0	0
MET	11	5	0	0	SDHC	3	1	0	0	MEN1	0	0	0	0
KDR	5	5	0	0	SMAD4	2	1	0	0	PTEN	0	0	0	0
CHEK2	5	5	2	2	CDKN2B	1	1	1	1	RAD51D	0	0	0	0
PDGFRA	5	5	0	0	MITF	1	1	0	0	RAS	0	0	0	0
RAD50	9	5	1	1	RAD51C	1	1	0	0	SDHAF2	0	0	0	0
BARD1	29	5	0	0	RET	2	1	0	0	SDHD	0	0	0	0
TSC1	5	5	0	0	BMPR1A	1	1	0	0	SMARCB1	0	0	0	0
PTCH1	5	5	0	0	CDK4	1	1	0	0	STK11	0	0	0	0
PALB2	5	4	0	0	CDKN2A	1	1	0	0	TP53	0	0	0	0
JAK2	6	4	0	0	MLH1	1	1	0	0					
FAM175A	4	4	0	0	NF2	1	1	0	0					

* Additional *BRCA* variants after excluding the primary diagnostic deleterious variant

"Potentially pathogenic" is defined as a nonsynonymous variant with a frequency <1% in both the Exome Variant Server (ESP6500) and HapMap control datasets

LoF, loss-of-function nonsynonymous variants defined as SNVs predicted to create a premature truncation (nonsense, disrupt, misstart or nontstop) and indels resulting in a frameshift

Table S10. Number of potentially pathogenic variants in disease genes (other than cancer) in the *BRCA1/2-* cohort

Gene	All Variants		LoF Variants		Gene	All Variants		LoF Variants		Gene	All Variants		LoF Variants	
	Cases	Variants	Cases	Variants		Cases	Variants	Cases	Variants		Cases	Variants	Cases	Variants
APOB	22	19	0	0	COL3A1	8	4	0	0	HFE2	2	2	0	0
CFTR	28	19	1	1	HMBS	4	4	0	0	KCNJ2	2	2	0	0
RYR1	21	17	1	1	LDLR	4	4	0	0	MYL2	2	2	1	1
CACNA1S	84	16	1	1	PCSK9	4	4	1	1	PRKAG2	2	2	0	0
DMD	19	14	0	0	TMEM43	8	4	0	0	TGFBR1	2	2	0	0
DSP	15	13	0	0	LDLRAP1	4	4	1	1	IDUA	1	1	1	1
ATP7B	10	12	0	0	KCNH2	6	3	0	0	OTC	19	1	0	0
HTT	44	11	0	0	HIP1	4	3	0	0	KCNE3	6	1	0	0
F5	20	11	1	1	SLC25A13	3	3	0	0	KCNE2	1	1	0	0
FBN1	10	9	0	0	DSC2	6	3	4	1	KCNE1	1	1	0	0
MYBPC3	11	8	0	0	SERPINC1	5	3	0	0	LMNA	1	1	0	0
MYH11	9	8	0	0	KCNQ1	4	3	0	0	MYL3	1	1	0	0
PKP2	12	8	0	0	CASQ2	3	3	0	0	QDPR	1	1	0	0
MYLK	13	8	0	0	COQ2	3	3	0	0	SCN1B	1	1	0	0
BCHE	8	8	0	0	TGFB3	3	3	0	0	TNNI3	1	1	0	0
RYR2	7	7	0	0	TGFBR2	3	3	0	0	PTS	1	1	1	1
SLC7A9	6	7	1	1	HOXB1	7	3	0	0	SGCD	1	1	0	0
SCN5A	8	6	0	0	PAH	2	2	0	0	TNNT2	0	0	0	0
CACNA1C	43	6	0	0	CACNB2	4	2	0	0	ACTA2	0	0	0	0
RBM20	8	6	0	0	KCNH1	2	2	0	0	ACTC1	0	0	0	0
DSG2	6	6	0	0	CPT2	4	2	0	0	ENG	0	0	0	0
PRSS1	8	5	0	0	CNBP	2	2	0	0	GCH1	0	0	0	0
HCN4	19	5	0	0	EMD	2	2	0	0	PCBD1	0	0	0	0
SLC37A4	6	5	1	1	HFE	8	2	0	0	PLN	0	0	0	0
GAA	5	5	1	1	COQ9	5	2	0	0	PRKAR1A	0	0	0	0
DMPK	5	5	0	0	PROS1	5	2	0	0	PROC	0	0	0	0
MYH7	5	4	0	0	HAMP	2	2	0	0	RBBP8	0	0	0	0
HIP1R	4	4	0	0	ACVRL1	2	2	0	0	SCN3B	0	0	0	0
INPP5B	4	4	2	2	GLA	2	2	0	0	SMAD3	0	0	0	0
SERPINA1	12	4	0	0	GPD1L	2	2	1	1	TPM1	0	0	0	0

"Potentially pathogenic" is defined as a nonsynonymous variant with a frequency <1% in both the Exome Variant Server (ESP6500) and HapMap control datasets
LoF, loss-of-function nonsynonymous variants defined as SNVs predicted to create a premature truncation (nonsense, disrupt, misstart or nontstop) and indels resulting in a frameshift

Table S11. Number of potentially pathogenic variants in cancer-associated genes for *BRCA1* - and *BRCA2* -mutant cohorts

Gene	BRCA1				BRCA2			
	All Variants		LoF Variants		All Variants		LoF Variants	
	Cases	Variants	Cases	Variants	Cases	Variants	Cases	Variants
APC	8	6	0	0	6	6	0	0
ATM	5	6	1	1	6	5	1	1
ATR	7	5	0	0	2	2	0	0
BAP1	0	0	0	0	1	1	0	0
BARD1	9	4	0	0	6	2	0	0
BLM	1	1	0	0	2	2	0	0
BMPR1A	0	0	0	0	1	1	0	0
BRCA1*	16	7	0	0	6	5	0	0
BRCA2*	15	11	0	0	16	9	0	0
BRIP1	0	0	0	0	0	0	0	0
CDC73	0	0	0	0	0	0	0	0
CDH1	2	2	0	0	1	1	0	0
CDK4	1	1	0	0	0	0	0	0
CDKN1A	0	0	0	0	0	0	0	0
CDKN2A	1	1	0	0	0	0	0	0
CDKN2B	0	0	0	0	0	0	0	0
CHEK1	0	0	0	0	0	0	0	0
CHEK2	1	1	1	1	1	1	0	0
CREBBP	3	3	0	0	3	3	0	0
EGFR	0	0	0	0	0	0	0	0
ELAC2	0	0	0	0	0	0	0	0
EPCAM	1	1	0	0	0	0	0	0
FAM175A	1	1	0	0	0	0	0	0
FH	0	0	0	0	0	0	0	0
FLCN	0	0	0	0	2	2	0	0
GALNT12	0	0	0	0	1	1	0	0
GEN1	10	1	10	1	17	2	16	1
GREM1	0	0	0	0	2	2	0	0
HOXB13	0	0	0	0	0	0	0	0
JAK2	2	2	0	0	1	1	0	0
KDR	1	1	0	0	2	2	0	0
KIT	0	0	0	0	0	0	0	0
MEN1	0	0	0	0	0	0	0	0
MET	2	2	0	0	6	4	0	0
MITF	0	0	0	0	0	0	0	0
MLH1	1	1	0	0	0	0	0	0
MLH3	3	3	0	0	1	1	0	0
MRE11A	2	2	0	0	1	1	0	0
MSH2	2	2	0	0	5	3	0	0
MSH6	5	4	0	0	1	1	0	0
MUTYH	1	1	0	0	0	0	0	0
NBN	0	0	0	0	1	1	0	0
NF1	2	1	0	0	1	1	0	0
NF2	0	0	0	0	1	1	0	0
NTRK1	2	3	0	0	0	0	0	0
PALB2	1	1	0	0	4	3	0	0
PDGFRA	2	2	0	0	2	2	0	0

* Additional *BRCA* variants after excluding the primary diagnostic deleterious variant

"Potentially pathogenic" is defined as a nonsynonymous variant with a frequency <1% in both the Exome Variant Server (ESP6500) and HapMap control datasets

Table S11 continued

Gene	BRCA1				BRCA2			
	All Variants		LoF Variants		All Variants		LoF Variants	
	Cases	Variants	Cases	Variants	Cases	Variants	Cases	Variants
PMS2	6	4	0	0	3	2	0	0
PTCH1	1	1	0	0	1	1	0	0
PTEN	0	0	0	0	0	0	0	0
RAD50	4	3	0	0	4	3	1	1
RAD51	2	2	0	0	0	0	0	0
RAD51C	0	0	0	0	0	0	0	0
RAD51D	0	0	0	0	0	0	0	0
RAS	0	0	0	0	0	0	0	0
RB1	3	3	0	0	1	1	0	0
RET	2	1	0	0	0	0	0	0
SDHAF2	0	0	0	0	0	0	0	0
SDHB	2	2	0	0	0	0	0	0
SDHC	1	1	0	0	1	1	0	0
SDHD	0	0	0	0	0	0	0	0
SMAD4	1	1	0	0	1	1	0	0
SMARCB1	0	0	0	0	0	0	0	0
SMO	2	2	0	0	5	4	0	0
STK11	0	0	0	0	0	0	0	0
TP53	0	0	0	0	0	0	0	0
TP53BP1	6	5	0	0	5	5	0	0
TSC1	0	0	0	0	2	2	0	0
TSC2	3	3	0	0	4	4	0	0
VHL	0	0	0	0	0	0	0	0
WT1	0	0	0	0	0	0	0	0
XRCC2	0	0	0	0	0	0	0	0
XRCC3	0	0	0	0	0	0	0	0

* Additional *BRCA* variants after excluding the primary diagnostic deleterious variant

"Potentially pathogenic" is defined as a nonsynonymous variant with a frequency <1% in both the Exome Variant Server (ESP6500) and HapMap control datasets

Table S12. Number of potentially pathogenic variants in disease genes (other than cancer) for *BRCA1* - and *BRCA2* -mutant cohorts

Gene	BRCA1				BRCA2			
	All Variants		LoF Variants		All Variants		LoF Variants	
	Cases	Variants	Cases	Variants	Cases	Variants	Cases	Variants
ACTA2	0	0	0	0	0	0	0	0
ACTC1	0	0	0	0	0	0	0	0
ACVRL1	1	1	0	0	1	1	0	0
APOB	8	7	0	0	4	4	0	0
ATP7B	1	1	0	0	5	7	0	0
BCHE	2	2	0	0	2	3	0	0
CACNA1C	14	3	0	0	14	4	0	0
CACNA1S	23	7	0	0	33	7	0	0
CACNB2	1	1	0	0	0	0	0	0
CASQ2	1	1	0	0	0	0	0	0
CFTR	7	7	0	0	10	8	0	0
CNBP	0	0	0	0	1	1	0	0
COL3A1	2	1	0	0	3	2	0	0
COQ2	2	2	0	0	0	0	0	0
COQ9	1	1	0	0	2	2	0	0
CPT2	1	1	0	0	2	2	0	0
DMD	5	4	0	0	7	7	0	0
DMPK	0	0	0	0	5	5	0	0
DSC2	1	1	0	0	4	3	3	1
DSG2	2	2	0	0	2	2	0	0
DSP	7	7	0	0	4	4	0	0
EMD	1	1	0	0	0	0	0	0
ENG	0	0	0	0	0	0	0	0
F5	7	4	0	0	4	4	0	0
FBN1	3	3	0	0	4	4	0	0
GAA	2	2	0	0	3	3	1	1
GCH1	0	0	0	0	0	0	0	0
GLA	0	0	0	0	1	1	0	0
GPD1L	0	0	0	0	0	0	0	0
HAMP	0	0	0	0	1	1	0	0
HCN4	0	0	0	0	13	4	0	0
HFE	2	1	0	0	4	2	0	0
HFE2	1	1	0	0	0	0	0	0
HIP1	2	2	0	0	2	2	0	0
HIP1R	1	1	0	0	1	1	0	0
HMBS	3	3	0	0	1	1	0	0
HOXB1	4	2	0	0	3	2	0	0
HTT	17	6	0	0	12	4	0	0
IDUA	0	0	0	0	1	1	1	1
INPP5B	1	1	1	1	1	1	0	0
KCNE1	0	0	0	0	1	1	0	0
KCNE2	0	0	0	0	1	1	0	0
KCNE3	3	1	0	0	2	1	0	0
KCNH1	0	0	0	0	0	0	0	0
KCNH2	2	2	0	0	1	1	0	0
KCNJ2	1	1	0	0	1	1	0	0
KCNQ1	1	1	0	0	2	2	0	0
LDLR	2	2	0	0	1	1	0	0

"Potentially pathogenic" is defined as a nonsynonymous variant with a frequency <1% in both the Exome Variant Server (ESP6500) and HapMap control datasets

Table S12 continued

Gene	BRCA1				BRCA2			
	All Variants		LoF Variants		All Variants		LoF Variants	
	Cases	Variants	Cases	Variants	Cases	Variants	Cases	Variants
LDLRAP1	2	2	0	0	1	1	1	1
LMNA	0	0	0	0	0	0	0	0
MYBPC3	4	4	0	0	2	2	0	0
MYH11	6	5	0	0	3	4	0	0
MYH7	1	2	0	0	2	2	0	0
MYL2	1	1	0	0	0	0	0	0
MYL3	0	0	0	0	0	0	0	0
MYLK	7	5	0	0	6	4	0	0
OTC	5	1	0	0	8	1	0	0
PAH	2	2	0	0	0	0	0	0
PCBD1	0	0	0	0	0	0	0	0
PCSK9	1	1	0	0	2	2	1	1
PKP2	3	3	0	0	4	3	0	0
PLN	0	0	0	0	0	0	0	0
PRKAG2	0	0	0	0	2	2	0	0
PRKAR1A	0	0	0	0	0	0	0	0
PROC	0	0	0	0	0	0	0	0
PROS1	2	1	0	0	1	1	0	0
PRSS1	5	4	0	0	3	3	0	0
PTS	1	1	1	1	0	0	0	0
QDPR	0	0	0	0	0	0	0	0
RBBP8	0	0	0	0	0	0	0	0
RBM20	1	1	0	0	3	3	0	0
RYR1	12	12	1	1	4	3	0	0
RYR2	4	4	0	0	2	2	0	0
SCN1B	1	1	0	0	0	0	0	0
SCN3B	0	0	0	0	0	0	0	0
SCN5A	4	4	0	0	1	1	0	0
SERPINC1	3	3	0	0	1	1	0	0
SGCD	0	0	0	0	0	0	0	0
SLC25A13	0	0	0	0	0	0	0	0
SLC37A4	1	1	0	0	2	2	0	0
SLC7A9	2	2	1	1	1	1	0	0
SMAD3	0	0	0	0	0	0	0	0
TGFB3	1	1	0	0	2	2	0	0
TGFBR1	0	0	0	0	1	1	0	0
TGFBR2	2	2	0	0	0	0	0	0
TMEM43	3	1	0	0	0	0	0	0
TNNI3	0	0	0	0	0	0	0	0
TNNT2	0	0	0	0	0	0	0	0
TPM1	0	0	0	0	0	0	0	0

"Potentially pathogenic" is defined as a nonsynonymous variant with a frequency <1% in both the Exome Variant Server (ESP6500) and HapMap control datasets

Table S13. Performance of WGS Variance Analysis

	PPV Allele Frequency Threshold	Heterozygous Rate Threshold	Interpretable Genes (%)		
			All PPVs	LoF PPVs	LoF SNV PPVs
**163 Disease-Gene Panel**	1%	1%	31.7	96.9	na
	**1%**	**2%**	**52.8**	**98.8**	na
	0.50%	1%	32.9	96.9	na
	0.10%	1%	42.2	96.9	na
	0.10%	0.20%	21.7	87.6	na
**ClinVar**	1%	1%	39.8	96.4	98.5
	**1%**	**2%**	**60.0**	**98.6**	**99.6**
	0.50%	1%	40.6	96.4	98.5
	0.10%	1%	46.5	96.8	98.8
	0.10%	0.20%	27.6	88.5	93.6

Interpretable genes = genes with <2% of the *BRCA1/2* cohort reporting PPVs

LoF = Loss-of-Function (nonsense, splice-site, frameshift, nonstop, misstart)

PPV = predicted pathogenic variant

SNV = single nucleotide variant

Table S14. Potentially pathogenic LoF variants in disease genes (other than cancer) in *BRCA1/2-* mutant individuals

Patient	Gene	Variant*	Pred Dz	Clinical Hx
Austosomal Dominant				
BRCA1.42	DSC2	**p.Ala897Lysfs*4**	ARVC	N.E.
BRCA1.87	RYR1	c.9001-2A>G	MH	N.E.
BRCA2.13	DSC2	**p.Ala897Lysfs*4**	ARVC	N.D.
BRCA2.14	DSC2	**p.Ala897Lysfs*4**	ARVC	N.D.
BRCA2.67	GPD1L	c.505+2T>G	BRGDA2	N.D.
BRCA2.71	DSC2	**p.Ala897Lysfs*4**	ARVC	N.D.
BRCA2.84	PCSK9	c.1863+1G>A	FH	N.D.
BRCA2.98	CACNA1S	**p.Met1828Argfs*71**	MH	N.E.
BRCA2.101	MYL2	p.Lys62*	HCM	N.E.
Austosomal Recessive				
BRCA1.10	PTS	p.Leu26*	BH4D	--
BRCA1.26	INPP5B	p.Arg859*	LS	--
BRCA1.27	CFTR	p.Gly542*	CF	--
BRCA1.66	INPP5B	p.Asn163*	LS	--
BRCA1.105	SLC7A9	p.Asn206Glufs*3	CSNU	--
BRCA2.21	SLC37A4	p.Cys243*	GSDIb	--
BRCA2.28	LDLRAP1	**p.His144Glnfs*27**	FH	--
BRCA2.58	GAA	**p.Pro238Argfs*31**	GSDII	--
BRCA2.85	IDUA	p.Trp402*	MPS I	--
BRCA2.95	F5	**p.Gly903Valfs*11**	FV	--

Abbreviations: ARVC = Arrhythmogenic right ventricular cardiomyopathy; BH4D = Tetrahydrobiopterin deficiency; BRGDA2 = Brugada syndrome-2; CF = Cystic fibrosis; CSNU = Cystinuria; FH = Familial hypercholesterolemia; FV = Factor V deficiency; GSDIb = Glycogen storage disease type Ib; GSDII = Glycogen storage disease, type II; HCM = Hypertrophic cardiomyopathy; LS = Lowe syndrome; MH = Malignant hyperthermia; MPS I = Mucopolysaccharidosis type I

* Indel variants are shown in bold

N.D., no data available on non-cancer personal or family history

N.E., no evidence in the available personal and family history

-- disease not expected due to heterozygosity

Table S15. Number of potentially pathogenic variants in cancer-associated genes in the non-*BRCA* cohort

Gene	All Variants		LoF Variants		Gene	All Variants		LoF Variants		Gene	All Variants		LoF Variants	
	Cases	Variants	Cases	Variants		Cases	Variants	Cases	Variants		Cases	Variants	Cases	Variants
BRCA2	15	15	0	0	PTCH1	1	2	0	0	SDHC	0	0	0	0
APC	14	12	3	3	FLCN	2	2	0	0	SMAD4	0	0	0	0
ATM	10	10	1	1	GEN1	10	2	9	1	CDKN2B	0	0	0	0
PALB2	9	7	2	2	EGFR	2	2	0	0	MITF	0	0	0	0
MSH6	9	7	3	3	RAD51C	2	2	1	1	NBN	0	0	0	0
TSC2	5	5	0	0	CDKN2A	2	2	0	0	SDHB	0	0	0	0
ATR	7	5	0	0	CHEK1	2	2	0	0	BMPR1A	0	0	0	0
TP53BP1	5	5	0	0	ELAC2	2	2	0	0	CDK4	0	0	0	0
MET	7	5	1	1	MLH3	1	1	0	0	CDKN1A	0	0	0	0
SMO	7	5	0	0	RB1	1	1	0	0	KIT	0	0	0	0
MUTYH	4	5	1	1	TSC1	1	1	0	0	NF2	0	0	0	0
RET	5	5	0	0	BLM	1	1	0	0	VHL	0	0	0	0
MSH2	4	4	1	1	CDH1	1	1	0	0	WT1	0	0	0	0
KDR	4	4	0	0	FAM175A	1	1	0	0	XRCC2	0	0	0	0
RAD50	4	4	1	1	EPCAM	1	1	0	0	BRIP1	0	0	0	0
MLH1	4	4	0	0	GREM1	2	1	0	0	CDC73	0	0	0	0
BRCA1	5	4	0	0	RAD51	1	1	0	0	HOXB13	0	0	0	0
PMS2	4	3	0	0	XRCC3	1	1	0	0	PTEN	0	0	0	0
CREBBP	5	3	0	0	FH	2	1	2	1	RAD51D	0	0	0	0
CHEK2	3	3	0	0	MEN1	1	1	0	0	RAS	0	0	0	0
PDGFRA	3	3	0	0	SDHAF2	2	1	0	0	SDHD	0	0	0	0
BARD1	22	3	0	0	TP53	1	1	1	1	SMARCB1	0	0	0	0
NF1	4	3	0	0	BAP1	0	0	0	0	STK11	0	0	0	0
MRE11A	2	2	0	0	NTRK1	0	0	0	0					
JAK2	2	2	0	0	GALNT12	0	0	0	0					

"Potentially pathogenic" is defined as a nonsynonymous variant with a frequency <1% in both the Exome Variant Server (ESP6500) and HapMap control datasets
LoF, loss-of-function nonsynonymous variants defined as SNVs predicted to create a premature truncation (nonsense, disrupt, misstart or nontstop) and indels resulting in a frameshift

Table S16. Number of potentially pathogenic variants in disease genes (other than cancer) in the non-*BRCA* cohort

Gene	All Variants		LoF Variants		Gene	All Variants		LoF Variants		Gene	All Variants		LoF Variants	
	Cases	Variants	Cases	Variants		Cases	Variants	Cases	Variants		Cases	Variants	Cases	Variants
APOB	14	15	0	0	LDLR	2	2	0	0	FBN1	0	0	0	0
HTT	26	11	1	1	IDUA	2	2	0	0	SLC37A4	0	0	0	0
**RYR1**	12	10	**1**	**1**	CPT2	2	2	0	0	KCNH2	0	0	0	0
CFTR	11	10	0	0	KCNQ1	2	2	0	0	HIP1	0	0	0	0
ATP7B	9	8	0	0	TGFB3	2	2	0	0	HMBS	0	0	0	0
DMD	8	7	0	0	TGFBR2	2	2	0	0	SERPINC1	0	0	0	0
CACNA1S	43	7	0	0	COQ9	2	2	0	0	COQ2	0	0	0	0
F5	7	7	0	0	PROS1	3	2	0	0	EMD	0	0	0	0
HCN4	5	6	0	0	KCNE3	3	2	0	0	ACVRL1	0	0	0	0
MYBPC3	6	6	0	0	MYH11	2	1	0	0	GPD1L	0	0	0	0
PKP2	6	6	0	0	BCHE	1	1	0	0	HFE2	0	0	0	0
SCN5A	7	6	0	0	SLC7A9	1	1	0	0	KCNJ2	2	0	0	0
RBM20	5	6	0	0	MYH7	1	1	0	0	PRKAG2	0	0	0	0
COL3A1	9	6	0	0	KCNH1	1	1	0	0	TNNT2	0	0	0	0
CACNA1C	30	5	0	0	SLC25A13	1	1	0	0	LMNA	0	0	0	0
DMPK	5	5	0	0	TMEM43	3	1	0	0	MYL3	0	0	0	0
DSP	4	4	0	0	CASQ2	1	1	0	0	QDPR	0	0	0	0
RYR2	4	4	0	0	CNBP	1	1	0	0	SCN1B	0	0	0	0
GAA	4	4	0	0	OTC	4	1	0	0	ACTA2	0	0	0	0
INPP5B	4	4	1	1	HFE	4	1	0	0	ACTC1	0	0	0	0
**DSC2**	5	4	**1**	**1**	HAMP	1	1	0	0	ENG	0	0	0	0
MYLK	8	3	0	0	GLA	1	1	0	0	GCH1	0	0	0	0
PAH	3	3	0	0	MYL2	1	1	0	0	PCBD1	0	0	0	0
SERPINA1	7	3	0	0	TGFBR1	1	1	0	0	PLN	0	0	0	0
**PCSK9**	3	3	**1**	**1**	KCNE2	1	1	0	0	PROC	0	0	0	0
LDLRAP1	3	3	0	0	HOXB1	3	1	0	0	PTS	0	0	0	0
PRSS1	2	2	0	0	KCNE1	1	1	0	0	SCN3B	0	0	0	0
**DSG2**	2	2	**1**	**1**	TNNI3	1	1	0	0	SGCD	0	0	0	0
HIP1R	2	2	0	0	PRKAR1A	1	1	0	0	SMAD3	0	0	0	0
CACNB2	4	2	0	0	RBBP8	1	1	0	0	TPM1	0	0	0	0

"Potentially pathogenic" is defined as a nonsynonymous variant with a frequency <1% in both the Exome Variant Server (ESP6500) and HapMap control datasets

LoF, loss-of-function nonsynonymous variants defined as SNVs predicted to create a premature truncation (nonsense, disrupt, misstart or nontstop) and indels resulting in a frameshift

bold = these patients' cardiac history did not reflect cardiomyopathy or in the case of *PCSK9* cholesterol was normal

Table S17. Potentially pathogenic LoF SNVs in autosomal dominant ClinVar genes in non-*BRCA* cancer genetics individuals

Patient	Gene	Variant	Predicted Autosomal Dominant Disease
UTSW1	PSTPIP1	c.985+2T>C	Pyogenic arthritis, pyoderma gangrenosum and acne
UTSW3	TNFRSF13B	p.Ser144*	Immunoglobulin A deficiency 2
UTSW3	HLA-G	c.2T>C	Asthma, susceptibility to
UTSW5	MIB1	p.Arg126*	Left ventricular noncompaction 7
UTSW9	HLA-G	c.74-2A>G	Asthma, susceptibility to
UTSW10	KRT83	p.Cys222*	Monilethrix
UTSW11	MASTL	p.Gln709*	Thrombocytopenia 2
UTSW11	FLG	p.Arg1474*	Ichthyosis vulgaris
UTSW12	KRT83	p.Cys222*	Monilethrix
UTSW13	IGF2	c.-3-1G>A	Russell-Silver syndrome
UTSW15	ANKRD26	c.3G>A	Thrombocytopenia 2
UTSW16	GJA8	p.Gln116*	Cataract 1
UTSW16	IGF2	c.-3-1G>A	Russell-Silver syndrome
UTSW16	TNXB	p.Glu316*	Ehlers-Danlos syndrome, type 3
UTSW18	TRPM4	c.2778+1G>A	Progressive familial heart block type 1B
UTSW21	PRPF8	p.Arg663*	Retinitis pigmentosa 13
UTSW23	KRT83	p.Cys222*	Monilethrix
UTSW27	TNFRSF11A	c.730+1G>T	Osteoporosis 7; Paget disease of bone
UTSW30	PRKCSH	c.-77-2A>C	Congenital cystic disease of liver
UTSW31	CLCN2	c.898+1G>A	Epilepsy with grand mal seizures on awakening
UTSW36	GUCA1B	c.207+1G>A	Retinitis pigmentosa 48
UTSW36	SLC16A12	c.-186-2A>T	Cataract, juvenile, with microcornea and glucosuria
UTSW37	LRRK2	p.Cys1313*	Parkinson disease 8
UTSW38	IGF2	c.-3-1G>A	Russell-Silver syndrome
UTSW40	TTN	c.30812-1G>A	Familial hypertrophic cardiomyopathy 9
UTSW40	PADI4	c.1A>G	Rheumatoid arthritis
UTSW41	HABP2	p.Arg177*	Thrombophilia; Deep vein thrombosis
UTSW42	SLC16A12	c.-182-2A>T	Cataract, juvenile, with microcornea and glucosuria
UTSW44	FLG	p.Arg501*	Ichthyosis vulgaris
UTSW45	SCN2A	c.-52+1G>T	Benign familial neonatal-infantile seizures
UTSW51	NAT2	c.-7+2T>C	Slow acetylator due to N-acetyltransferase enzyme variant
UTSW52	NAT2	c.-7+2T>C	Slow acetylator due to N-acetyltransferase enzyme variant
UTSW53	BCMO1	c.1102-1G>A	Vitamin a deficiency
UTSW55	NME1	c.-149-1G>C	Neuroblastoma
UTSW57	CYP3A5	p.Leu249*	Essential hypertension
UTSW64	COL9A2	c.303+2T>C	Multiple epiphyseal dysplasia 2
UTSW67	PDE11A	p.Arg101*	Nodular adrenocortical disease, primary; Cushing syndrome
UTSW69	LDB3	p.Arg219*	Dilated cardiomyopathy; Myofibrillar myopathy
UTSW70	KRT83	p.Cys222*	Monilethrix
UTSW70	MIB1	p.Arg676*	Left ventricular noncompaction 7
UTSW72	AGBL1	p.Arg1028*	Corneal dystrophy, fuchs endothelial, 8
UTSW73	PRKCSH	c.-77-2A>C	Congenital cystic disease of liver
UTSW78	MET	p.Arg1148*	Hepatocellular carcinoma; RCC, papillary
UTSW80	CALCR	c.-43-2A>G	Osteoporosis
UTSW80	IGF2	p.Gln33*	Russell-Silver syndrome
UTSW84	FLG	p.Arg501*	Ichthyosis vulgaris

Table S18. Potentially pathogenic LoF SNVs in autosomal recessive and X-linked ClinVar genes in non-*BRCA* cancer genetics individuals

Patient	Gene	Variant	Predicted Disease
Autosomal Recessive			
UTSW1	ACSF3	c.823-2A>G	Combined malonic and methlmalonic aciduria
UTSW1	PINK1	p.Glu563*	Parkinson disease 6, early-onset
UTSW1	PNPLA6	p.Glu28*	Spastic paraplegia 39; Boucher Neuhauser syndrome
UTSW2	ACSF3	c.823-2A>G	Combined malonic and methlmalonic aciduria
UTSW4	SLC11A2	c.-39+2T>A	Hypochromic microcytic anemia with iron overload
UTSW6	MMACHC	p.*283Leu	Methylmalonic acidemia with homocystinuria
UTSW7	GYS2	p.Arg147*	Hypoglycemia with deficiency of glycogen synthetase in the liver
UTSW7	SEC23B	p.Arg217*	Congenital dyserythropoietic anemia, type II
UTSW8	LAMB3	c.565-1G>A	Epidermolysis bullosa
UTSW8	TMEM216	p.Arg85*	Joubert syndrome; Meckel syndrome
UTSW10	GJB6	c.-295-2A>G	Clouston syndrome; deafness
UTSW10	HAL	p.Arg356*	Histidinemia
UTSW10	HAVCR1	c.-13+1G>A	Ige responsiveness, atopic
UTSW10	SLC26A5	c.-53-2A>G	Deafness, 61
UTSW10	SYNE1	c.6071+1G>A	Spinocerebellar ataxia, 8
UTSW11	FLG	p.Arg1474*	Ichthyosis vulgaris
UTSW12	CEP57	p.Arg151*	Mosaic variegated aneuploidy syndrome 2
UTSW12	NBAS	c.4461+2T>C	Short stature, optic nerve atrophy, and pelger-huet anomaly
UTSW12	NPC2	c.441+1G>A	Niemann-Pick disease type C2
UTSW13	ARID1A	c.4102-2A>G	Coffin-Siris syndrome
UTSW13	CEP164	p.Arg4*	Nephronophthisis 15
UTSW13	GRIP1	p.Arg1055*	Fraser syndrome
UTSW13	RAD51C	p.Arg193*	Fanconi anemia, complementation group O
UTSW14	SLC26A5	c.-53-2A>G	Deafness, 61
UTSW16	CCDC65	p.Met1Lys	Ciliary dyskinesia, primary, 27
UTSW16	CEP135	p.Lys1071*	Primary microcephaly 8
UTSW17	ACSF3	c.823-2A>G	Combined malonic and methlmalonic aciduria
UTSW17	HEXA	c.1073+1G>A	Deafness, 61
UTSW18	SLC26A5	c.-53-2A>G	Deafness, 61
UTSW19	OTOA	p.Glu708*	Deafness, 22
UTSW19	PIGN	p.Glu61*	Multiple congenital anomalies-hypotonia-seizures syndrome
UTSW21	ASNS	p.Arg386*	Asparagine synthetase deficiency
UTSW21	CARD9	c.1434+1G>C	Candidiasis, familial, 2
UTSW21	TCTN1	p.Cys93*	Joubert syndrome 13
UTSW22	FANCM	p.Gln1701*	Fanconi anemia, complementation group M
UTSW24	AURKC	c.1+1G>A	Spermatogenic failure 5
UTSW24	WRN	p.Arg1406*	Werner syndrome
UTSW25	ARMC4	p.Trp359*	Primary ciliary dyskinesia 23
UTSW26	H6PD	p.Arg551*	Deficiency of (R)-20-hydroxysteroid dehydrogenase
UTSW27	TNFRSF11A	c.730+1G>T	Osteoporosis 7; Paget disease of bone
UTSW28	DMGDH	p.Trp324*	Dimethylglycine dehydrogenase deficiency
UTSW31	DMP1	c.55-2A>G	Hypophosphatemic vitamin D refractory rickets
UTSW32	PTPRQ	c.1366+2T>C	Deafness, 84
UTSW32	TCTN1	p.Met1Ile	Joubert syndrome 13
UTSW33	MGP	c.62-1G>A	Keutel syndrome
UTSW34	WNT10B	p.Arg360*	Split-hand/foot malformation 6
UTSW35	FA2H	p.Arg189*	Spastic paraplegia 35
UTSW35	TMPRSS6	p.Trp402*	Microcytic anemia
UTSW36	TCTN1	p.Met1Ile	Joubert syndrome 13
UTSW37	SLC27A4	c.1324+1G>T	Ichthyosis prematurity syndrome
UTSW38	SAMD9	p.Glu871*	Tumoral calcinosis, familial, normophosphatemic
UTSW39	NPHP4	p.Gln1392*	Nephronophthisis 4
UTSW40	LOXHD1	p.Arg383*	Deafness, 77
Autosomal Recessive continued			
UTSW43	GBE1	c.691+2T>C	Glycogen storage disease, type IV
UTSW44	FLG	p.Arg501*	Ichthyosis vulgaris
UTSW45	NHEJ1	c.529+1G>A	Severe combined immunodeficiency with microcephaly, growth retardation, and sensitivity to ionizing radiation
UTSW45	TCTN1	p.Met1Ile	Joubert syndrome 13
UTSW46	COL7A1	p.Arg137*	Dystrophic epidermolysis bullosa
UTSW49	DYSF	c.3349-2A>G	Limb-girdle muscular dystrophy
UTSW49	HAL	p.Trp280*	Histidinemia
UTSW51	RGS9	c.58-2A>G	Prolonged electroretinal response suppression
UTSW51	FANCM	pGln1701*	Fanconi anemia, complementation group M
UTSW52	ACSF3	c.823-2A>G	Combined malonic and methlmalonic aciduria
UTSW52	RGS9	c.58-2A>G	Prolonged electroretinal response suppression
UTSW53	KIF7	p.Glu529*	Acrocallosal syndrome, Schinzel type
UTSW54	CCDC39	c.609+1G>C	Ciliary dyskinesia, primary, 14
UTSW56	MTFMT	p.Arg332*	Combined oxidative phosphorylation deficiency 15
UTSW56	OTOG	p.Gln834*	Deafness, 18b
UTSW56	UNC13D	p.Arg899*	Hemophagocytic lymphohistiocytosis, familial, 3
UTSW57	C5orf42	c.8300-1G>C	Joubert syndrome 17
UTSW60	DPYD	c.1905+1G>A	Dihydropyrimidine dehydrogenase deficiency
UTSW62	B3GALTL	c.660+1G>A	Peters plus syndrome
UTSW64	RARS2	p.Met1Arg	Pontocerebellar hypoplasia type 6
UTSW64	COL9A2	c.303+2A>G	Multiple epiphyseal dysplasia 2
UTSW65	HPS5	p.Met1Ile	Hermansky-Pudlak syndrome 5
UTSW68	FOLR1	c.493+2T>C	Cerebral folate deficiency
UTSW70	DNAH11	c.5778+1G>A	Kartagener syndrome; Ciliary dyskinesia, primary, 7
UTSW71	HYDIN	c.3786-1G>T	Ciliary dyskinesia, primary, 5
UTSW73	CDK5RAP2	p.Gln1258*	Primary microcephaly 3
UTSW73	PCK2	p.Ser23*	Phosphoenolpyruvate carboxykinase deficiency, mitochondrial
UTSW75	CEP164	p.Gln1410*	Nephronophthisis 15
UTSW75	GBE1	p.Arg524*	Glycogen storage disease, type IV
UTSW75	GPR98	p.Gln2301*	Usher syndrome, type 2C
UTSW75	TPMT	p.Glu98*	Thiopurine methyltransferase deficiency
UTSW76	ERCC3	p.Arg109*	Trichothiodystrophy photosensitive
UTSW77	FUT6	p.Tyr315*	Fucosyltransferase 6 deficiency
UTSW77	ICK	p.Ser472*	Endocrine-cerebroosteodysplasia
UTSW78	CD36	p.Leu360*	Macro-thrombocytopenia; susceptibility to malaria
UTSW78	CHIT1	c.314+1G>T	Chitotriosidase deficiency; Gaucher disease
UTSW80	MYO3A	p.Arg1561*	Deafness, 30
UTSW81	HAVCR1	c.-13+1G>A	Ige responsiveness, atopic
UTSW81	LOXHD1	p.Arg383*	Deafness, 77
UTSW82	TCTN1	p.Met1Ile	Joubert syndrome 13
UTSW82	TPMT	p.Glu98*	Thiopurine methyltransferase deficiency
UTSW82	UNC13D	c.753+1G>T	Hemophagocytic lymphohistiocytosis, familial, 3
UTSW82	FANCA	p.Glu288*	Fanconi anemia, complementation group A
UTSW83	PEX6	c.882+1G>A	Peroxisome biogenesis disorders, Zellweger syndrome spectrum
UTSW84	FLG	p.Arg501*	Ichthyosis vulgaris
UTSW85	FIG4	p.Lys559*	Charcot-Marie-Tooth disease, type 4J
X-linked			
UTSW1	MED12	p.Gln1263*	Ohdo syndrome; FG Syndrome
UTSW13	MED12	p.Gln1263*	Ohdo syndrome; FG Syndrome
UTSW14	SLC16A2	p.Met1Leu	Allan-Herndon-Dudley syndrome
UTSW75	ALAS2	p.Met1Ile	Hereditary sideroblastic anemia
